# Intrinsic chicory root fibers modulate colonic microbial butyrate-producing pathways and improve insulin sensitivity in individuals with obesity

**DOI:** 10.1016/j.xcrm.2025.102237

**Published:** 2025-07-15

**Authors:** Lina Omary, Emanuel E. Canfora, Marie-Luise Puhlmann, Asimenia Gavriilidou, Iris Rijnaarts, Jens J. Holst, Yvonne M.H. Op den Kamp-Bruls, Willem M. de Vos, Ellen E. Blaak

**Affiliations:** 1 Department of Human Biology, NUTRIM Institute of Nutrition and Translational Research in Metabolism, Maastricht University Medical Center+, Maastricht, the Netherlands; 2 Laboratory of Microbiology, Wageningen University & Research, Wageningen, the Netherlands; 3 Division of Human Nutrition and Health, Wageningen University & Research, Wageningen, the Netherlands; 4 Department of Nutrition, Exercise & Sports, University of Copenhagen, Copenhagen, Denmark; 5 WholeFiber Holding BV, Emmeloord, the Netherlands; 6 Department of Biomedical Sciences, Novo Nordisk Foundation Center for Basic Metabolic Research, University of Copenhagen, Copenhagen, Denmark; 7 Department of Radiology and Nuclear Medicine, NUTRIM Institute of Nutrition and Translational Research in Metabolism, Maastricht University Medical Center+, Maastricht, the Netherlands; 8 Human Microbiome Research Program, Faculty of Medicine, University of Helsinki, Helsinki, Finland

**Keywords:** gut microbiota, butyrate, intrinsic fibers, diabetes, obesity, insulin sensitivity, adipocytes, interorgan crosstalk

## Abstract

Type 2 diabetes and obesity have become major public health concerns. Growing evidence suggests that increased dietary fiber intake, through its interaction with the gut microbiota, may help prevent these diseases. Here, we demonstrate in a 12-week randomized, placebo-controlled trial in individuals at risk for type 2 diabetes that intake of an intrinsic fiber product, consisting of entire plant cells, tended to improve peripheral insulin sensitivity (*p* = 0.085), increased whole-body insulin sensitivity (*p* = 0.032), reduced circulating triglycerides (*p* = 0.049), and tended to reduce intrahepatic lipid content (*p* = 0.063), along with an increased proportion of small adipocytes (*p* = 0.008). Phylogenetic and metagenomic analysis revealed that these outcomes coincided with increased levels of fiber-degrading *Bifidobacterium* spp. and butyrate-producing *Anaerostipes* spp. and a functional shift toward a distal butyrogenic trophic chain while the best responding individuals had increased levels of pectin degraders that may produce propionate. Our findings demonstrate the pivotal role of slowly fermented, intrinsic plant cell fibers in improving cardiometabolic health.

This study was registered at ClinicalTrials.gov (NCT04714944).

## Highlights

• 35 participants with insulin resistance participate in a 12-week intrinsic fiber trial
• Intrinsic chicory root fiber improves insulin sensitivity and cardiometabolic health
• This coincides with increased fecal butyrate- and propionate-producing taxa
• This offers a fresh view on slowly fermented plant fibers in enhancing cardiometabolic health

## Graphical abstract

**Figure:**
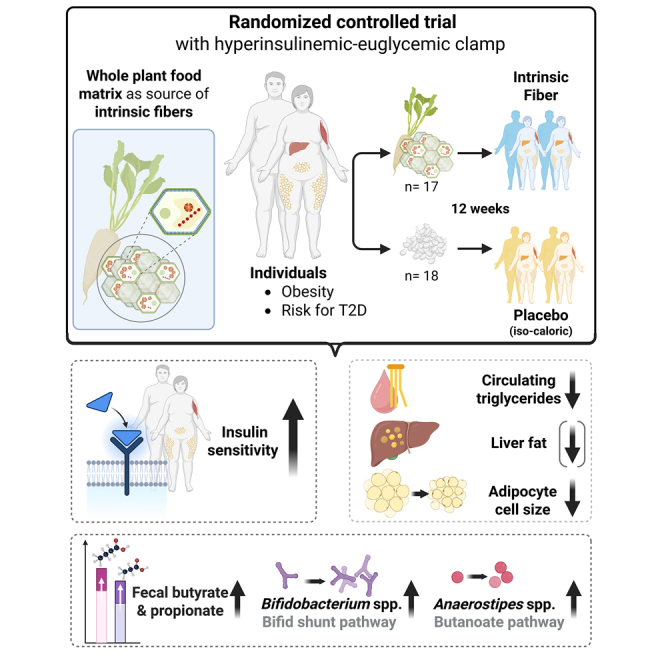

## Teaser

Omary et al. show that 12-week supplementation with intrinsic chicory root fiber improves insulin sensitivity and cardiometabolic health. These benefits are accompanied by a functional shift toward a distal colonic butyrogenic trophic chain and propionate-producing taxa, demonstrating a pivotal role of slowly fermented plant fibers in metabolic health.

## Introduction

The burgeoning global health crisis of obesity and related health risk, including insulin resistance (IR), type 2 diabetes (T2D), and cardiovascular disease, necessitates interventions, particularly those targeting the underlying metabolic dysfunctions.^1^ Growing evidence implicates the human gut microbiota, a complex ecosystem of primarily bacterial species residing in the gastrointestinal (GI) tract, as a central contributor in maintaining host gut and metabolic health.^2^ Disturbances in gut microbiota composition and its functionality may contribute to the development of obesity, IR, and T2D, by affecting energy and substrate metabolism, adipose tissue metabolism, and systemic and tissue inflammatory profile.^3^

The gut microbiome can influence metabolic processes through direct communication of end products derived from the fermentation of dietary fibers such as short-chain fatty acids (SCFAs) with metabolic active organs, including skeletal muscle, liver, and adipose tissue.^3^^,^^4^^,^^5^^,^^6^ Acetate, propionate, and butyrate are the most common and abundant SCFAs produced by mainly anaerobic bacteria^7^ and have significant roles in various physiological processes, including metabolic, endocrine, and immune regulation.^3^^,^^8^ In particular, a reduction in the abundance of butyrate-producing bacteria is associated with metabolic disturbances, including IR and increased inflammation.^9^ Additionally, the presence of these bacteria has been shown to influence glucose and lipid metabolism, suggesting their potential role in maintaining metabolic homeostasis and preventing the onset of T2D.^9^

The functional capabilities of the gut microbiota exhibit spatial variation along the colon, with the proximal colon identified as the primary site for saccharolytic fermentation, mainly due to the high availability of fermentable dietary fibers there.^10^ Interestingly, our previous research demonstrated that colonic infusions of mixtures of SCFAs, at concentrations and ratios reached after fiber intake, increased whole-body fat oxidation, energy expenditure, and the production of the satiety-stimulating hormone peptide YY (PYY) in men with overweight and obesity.^11^^,^^12^ Of note, this effect was observed when SCFAs were administered into the distal colon rather than the proximal colon.^12^ Similarly, rectal and not intravenous administration of acetate raised plasma PYY and Glucagon-like Peptide-1 (GLP-1) levels in females with hyperinsulinemia.^13^ This led to the hypothesis that an increase in distal colonic saccharolytic fermentation is a key modulating factor for the prevention of chronic metabolic diseases such as obesity and T2D.^14^^,^^15^

Yet, prevailing studies have often narrowly focused on isolated fiber sources and mixtures thereof, yielding limited and controversial effects on the microbiota and host metabolism.^16^ The study of isolated or purified dietary compounds has provided valuable mechanistic insight; however, it does not take into account that dietary fibers are either part of the plant cell wall (pectin, hemicellulose, and cellulose) or encapsulated by it (storage carbohydrates such as inulin), hence termed “intrinsic fibers.”^17^ Unlike isolated fibers, intrinsic fibers are unextracted and unmilled; thus, the fibers are contained within their original plant cell matrix, shielding them from immediate breakdown and influencing their physiological behavior and microbial degradation.^17^^,^^18^^,^^19^ This intrinsic structure may lead to a more gradual release of fibers utilized by the gut microbiota and a subsequent slow fermentation, thereby potentially increasing the availability of microbial substrates in the distal colon.^20^ By utilizing this preserved three-dimensional fiber organization in cubes of dried chicory roots, containing a high intrinsic fiber content (85%), particularly rich in inulin (70% inulin, 10% pectin, and 5% hemi-/cellulose),^18^^,^^21^ our approach sets itself apart from common reductionist trends, rendering our findings more broadly applicable and aligning with epidemiological evidence of dietary fiber health benefits,^22^ which have seldom been confirmed with isolated single fibers. Interestingly, we previously found that short-term (3 weeks), daily intake of 30 g/day of dried chicory root fibers in individuals at risk of T2D rapidly modulated the gut microbiota by increasing relative levels of *Bifidobacterium* spp. and *Anaerostipes* spp. by 3-fold that could form a butyrogenic trophic chain.^23^ This was accompanied by higher levels of fecal SCFAs, including a 20% increase in butyrate.^23^ Additionally, using fecal *in vitro* batch fermentations, we confirmed high production levels of SCFAs from the intrinsic fiber product, notably at a late stage during the fermentation, coinciding with the presence of pectin-degrading and butyrate-producing bacteria.^20^

**Figure 1:**
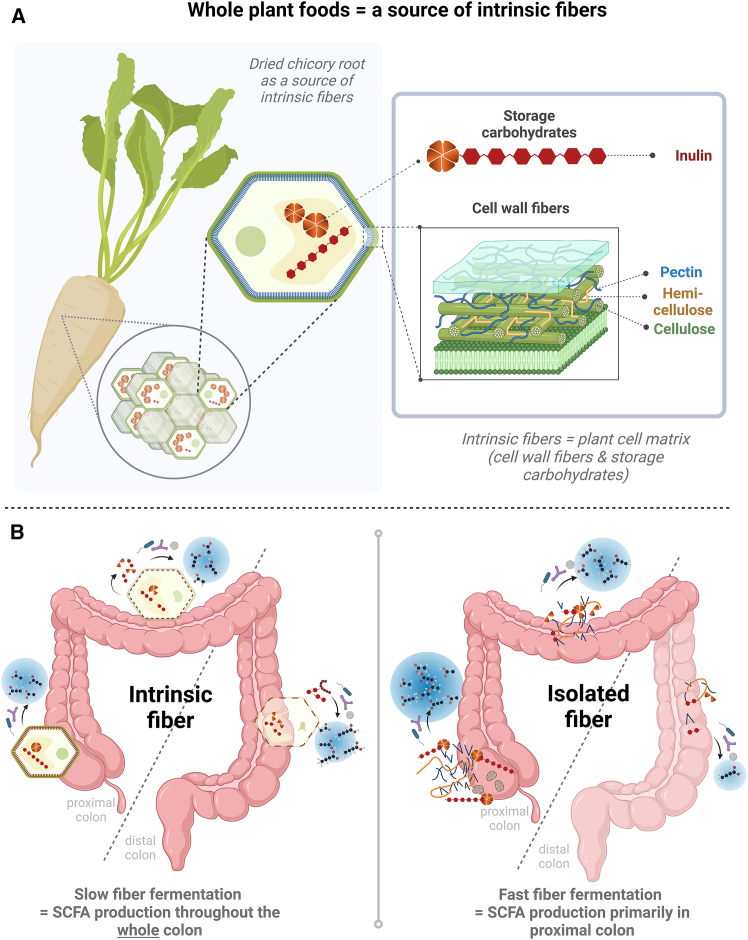
Our hypothesis is that longer-term consumption of intrinsic fibers, as present in the dried chicory root product (A) may lead to a more gradual release of fibers utilized by the gut microbiota. The subsequent slow fermentation stimulates the distal production of colonic SCFAs, in particular butyrate. (B) This is in contrast to rapidly fermentable isolated fibers, which produce SCFA mainly in the proximal colon. (C) An increase in distal colonic saccharolytic fermentation is a key modulating factor that might offer promising prevention strategies for chronic metabolic diseases such as obesity and T2D. Created with BioRender.com.

Here, we hypothesized that longer-term consumption of intrinsic fibers, as present in the dried chicory root product, stimulates the distal production of colonic SCFAs resulting thereby in improvements in peripheral insulin sensitivity and metabolic health (as depicted in Figure 1). We have employed a robust randomized controlled trial using this intrinsic fiber in individuals with obesity at high risk of developing T2D followed by state-of the-art phenotyping of metabolic health with a two-step hyperinsulinemic-euglycemic clamp to assess tissue-specific insulin sensitivity and magnetic resonance spectrometry (MRS) to measure liver fat accumulation, as well as dual X-ray absorptiometry (DEXA) scan, subcutaneous adipose tissue biopsies, and targeted metabolomics, which were combined with advanced gut microbiomics using phylogenetic profiling and deep metagenomic analysis.

Interestingly, we show a pivotal role of the intrinsic chicory root fibers in modulating microbial butyrate production pathways and fecal SCFA concentrations, as well as whole-body insulin sensitivity, insulin-mediated non-oxidative glucose disposal, fasting circulating glucose, and triglyceride (TG) concentrations with trends toward an increased peripheral insulin sensitivity and reduced liver fat accumulation. Additionally, we noticed a shift in adipocyte cell size toward smaller adipocytes, indicating an improved adipocyte function independent of any weight changes. We propose that fiber-derived microbial butyrate and propionate play a pivotal role in these improvements as we observe an inverse relation between improved metabolic health markers and increased fecal butyrate and propionate concentrations, particularly notable among participants responsive to the intervention. Our findings have important implications for dietary intervention strategies and guidelines in the prevention of T2D and cardiometabolic disease.

## Results

### Study design and participant characteristics

In this randomized, double-blind, placebo-controlled, parallel designed intervention study, 87 men and women aged 45–70 years with a body mass index (BMI) 28–35 kg/m^2^ were screened for eligibility. The volunteers were enrolled in the study if they were insulin resistant (homeostasis model assessment for insulin resistance, HOMA-IR > 2.2) and/or had prediabetes (according to American Diabetes Association criteria: fasting blood glucose >5.6 and <7.0 mmol/L or 2 h postprandial glucose after a 2 h oral glucose tolerance test [OGTT] >7.8 and <11.1 mmol/L.^24^ At screening, in addition, anthropometric measures and general health assessments were performed (Figure S1).

Forty-two participants were included and randomized into two groups between January 2021 and March 2023. One group was supplemented with an intrinsic fiber product (WholeFiber) consisting of cubes of dried chicory root (intrinsic fiber) for 12 weeks. The control group received an iso-caloric, low-fiber-containing placebo alternative from puffed millet, a round light brown grain, which had a comparable appearance and taste as dried chicory root (see Table S1 for nutritional composition of WholeFiber and puffed millet). The 12-week study period consisted of a 2-week run-in period with half the dosage and a 10-week period with the full dosage of 30 g/day intrinsic fiber or iso-caloric placebo. Throughout the study period, four participants dropped out in the intrinsic fiber group and three in the placebo group. Therefore, a total of 35 participants (*n* = 17 intrinsic fiber, *n* = 18 placebo) were included in the final analysis (Figure S2). No significant differences in baseline characteristics were present between groups (Table 1). Compliance was 96.3% and 98.1% for intrinsic fiber and placebo group, respectively. This was verified by counting returned empty sachets in addition to checking fiber intake log and cross-referenced against the number of days participants were undergoing investigation (Table S2). No serious adverse events occurred during the study. Adverse events experienced by participants that were not related to the study product or procedures are reported in Table S3.

**Table 1:** Participants’ baseline characteristics at screening (*n* = 35)

	Intrinsic fiber (*n* = 17)	Placebo (*n* = 18)	*p* value
Gender, male/female	10/7	10/8	–
Age, y	62 ± 6	63 ± 6	0.518
Weight, kg	98.02 ± 13.47	95.82 ± 15.57	0.659
Height, m	1.73 ± 0.09	1.72 ± 0.09	0.732
Body mass index, kg/m^2^	32.6 ± 4.4	32.1 ± 2.6	0.666
Waist-hip ratio	1.00 ± 0.08	0.97 ± 0.08	0.391
Systolic blood pressure, mm Hg	135 ± 17	131 ± 12	0.428
Diastolic blood pressure, mm Hg	88 ± 10	85 ± 7	0.191
Fasting glucose level, mmol/L	5.99 ± 0.56	5.91 ± 0.59	0.672
OGTT 2-h plasma glucose, mmol/L	7.58 ± 2.52	8.18 ± 2.50	0.480
Fasting insulin level, uU/mL	12.0 ± 5.9	13.3 ± 6.9	0.563
HOMA-IR	3.22 ± 1.77	3.56 ± 2.02	0.591
Hemoglobin A1c, %	5.68 ± 0.33	5.72 ± 0.27	0.696

Data are shown as mean ± SD. Data that were normally distributed were analyzed with the independent t test (age, height, blood pressure, glucose levels, and hemoglobin A1c), while not normally distributed data were analyzed using the Mann-Whitney U test (weight, BMI, waist-hip ratio, insulin, and HOMA-IR). HOMA-IR, homeostatic model assessment of insulin resistance; OGTT, oral glucose tolerance test.

### GI symptoms

GI-related outcomes were monitored using questionnaires that assessed various GI-related activities such as stool frequency, stool softness, and GI symptoms. The intrinsic fiber group experienced a mean increase by half a unit on the Bristol stool form scale and a median increase in stool frequency by one defecation per day after the intervention but did not reach statistical significance compared to placebo (Table S4). We observed no to minor changes in bloating, rumbling, cramping, or regurgitation over time in both the groups with only flatulence increasing significantly over time for the intrinsic fiber group (Friedman test: χ2(4) = 20.66, *p* < 0.001; Table S5) reaching the highest score at week 12 (paired Wilcoxon test *p* adjusted = 0.021; Table S3).

### Habitual physical activity and diet

No differences in activity levels or daily energy and macronutrient intake were observed between groups throughout the intervention period as assessed using a validated physical activity questionnaire (SQUASH [Short Questionnaire to Asess Health-Enhancing Physical Activity], Table S6) and a 3-day food diary recorded on 2 weekdays and 1 weekend day at baseline, week 6, and week 12 (Table S7).

### Body composition

Body composition analysis utilizing DEXA indicated no significant changes in total body weight, body fat percentage, absolute fat and lean body mass, or visceral fat mass between groups prior to the start of the study and after 12-week intervention (Table S8).

### Intrinsic fiber increases relative abundance of SCFA-producing bacterial species

**Figure 2:**
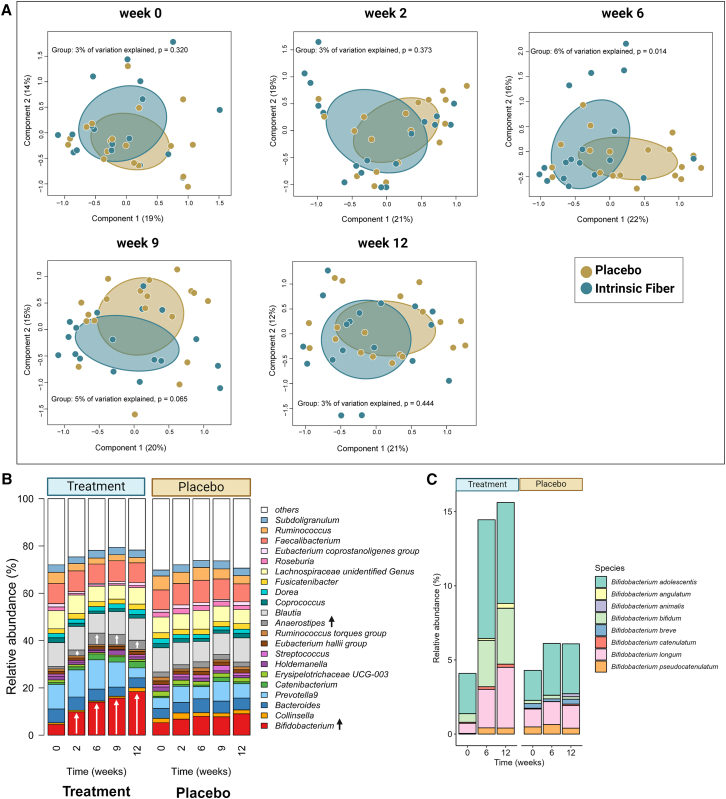
Modulation of the gut microbiota assessed from fecal samples over time by the intrinsic fiber product compared to placebo (A–C) Differences in fecal microbiota composition based on 16S rRNA amplicon sequencing assessed by β-diversity (A), representing here the between-sample differences, using Bray-Curtis dissimilarity over time (week 0, 2, 6, 9, and 12). Differences in β-diversity were calculated using permutational multivariate analysis of variance (PERMANOVA). (B) Common taxa (mean relative abundance of at least 1% and mean relative prevalence of at least 50% of all samples) based on 16S rRNA amplicon sequencing in the intrinsic fiber and the placebo group with statistically significant increases in *Bifidobacterium* spp. and *Anaerostipes* spp. over time after dried chicory root intake. (C) Changes in *Bifidobacterium* species relative abundances (%) based on taxonomic profiling using metagenomics in the placebo and intrinsic fiber group at weeks 0, 6, and 12. Created with BioRender.com.

The assessment of fecal microbiota composition and changes in the relative abundance of individual genera was done using 16S rRNA gene amplicon sequencing. Species-level microbiota changes and changes in functional profiling of microbial pathways and genes were subsequently determined using metagenomic sequence analysis at baseline, week 6, and week 12. At baseline, both groups had a similar fecal microbiota composition assessed by principal coordinate analysis (PCoA)-based β-diversity using Bray-Curtis dissimilarity (Figure 2A). We observed a strong modulatory effect of the intrinsic fiber product on fecal microbiota composition, which in this context reflects the between-group differences in gut microbiota composition. Following 2-week intrinsic fiber intake, fecal microbiota composition started to differ between groups and became significantly different from 6 weeks onwards (permutational multivariate analysis of variance [PERMANOVA] *p* = 0.014; Figure 2A), and this continued up until week nine (PERMANOVA *p* = 0.065; Figure 2A) before diminishing at week 12 (PERMANOVA *p* = 0.444; Figure 2A).

The changes in overall fecal microbiota composition were paralleled by changes in individual taxa that at genus level were primarily driven by differences in levels of *Bifidobacterium* spp. and *Anaerostipes* spp. (Figure 2B). The intrinsic fiber intake rapidly increased the mean relative abundances of *Bifidobacterium* spp. by 2.2-fold at 2 weeks (*p* = 0.013, *q* = 0.224), further increasing to 3.1-fold at week six (*p* < 0.001, *q* < 0.001) and 3.5-fold at week nine (*p* < 0.001, *q* < 0.001), resulting in a 4.1-fold increase at week 12 (*p* < 0.001, *q* < 0.001) compared to baseline (Table S9). Similarly, the mean relative abundances of *Anaerostipes* spp. increased rapidly within 2 weeks by 2.5-fold (*p* < 0.001, *q* < 0.001), peaking at week six with 4.8-fold higher levels (*p* < 0.001, *q* < 0.001) and remaining elevated at week nine with a 4.4-fold increase (*p* < 0.001, *q* < 0.001), before slightly decreasing to 3.7-fold elevation at 12 weeks (*p* < 0.001, *q* < 0.001; Table S10). In our previous work, using 16S rRNA gene amplicon taxonomic profiling, we detected predominantly *Bifidobacterium longum*, while *Anaerostipes* spp. mainly consisted of *Anaerostipes hadrus*, the most abundant in adults.^23^^,^^25^ Here, metagenome-based taxonomic profiling confirmed the presence of *Bifidobacterium longum* alongside *Bifidobacterium bifidum* and *Bifidobacterium adolescentis* (Figure 2C) as well as the exclusive presence of *Anaerostipes hadrus.* Several other genera, such as *Blautia* spp. and *Coprococcus* spp., decreased over time in the intrinsic fiber group, although none were statistically significant after multiple-testing correction. Changes in taxa at the genus level in the placebo group were minimal and inconsistent over time (Table S10). Overall, the present results confirm our earlier findings^23^ in participants at a different geographic location and with different intervention duration and highlight the consistent and reproducible effects of the intrinsic fiber intake on gut microbiota composition, particularly the 4-to 5-fold increase in relative abundances of bifidobacteria and *Anaerostipes* spp.

To explore the interactions between the gut microbiota and host metabolic processes, we next examined the shifts in plasma and fecal levels of SCFAs and microbial pathways via metagenomic functional profiling, stemming from the bacterial fermentation of dietary fiber. SCFAs are recognized as crucial agents facilitating the two-way communication between gut bacteria and the host’s metabolic organs.

### Intrinsic fiber intake increases microbial butyrate production pathways and fecal SCFA concentrations

**Figure 3:**
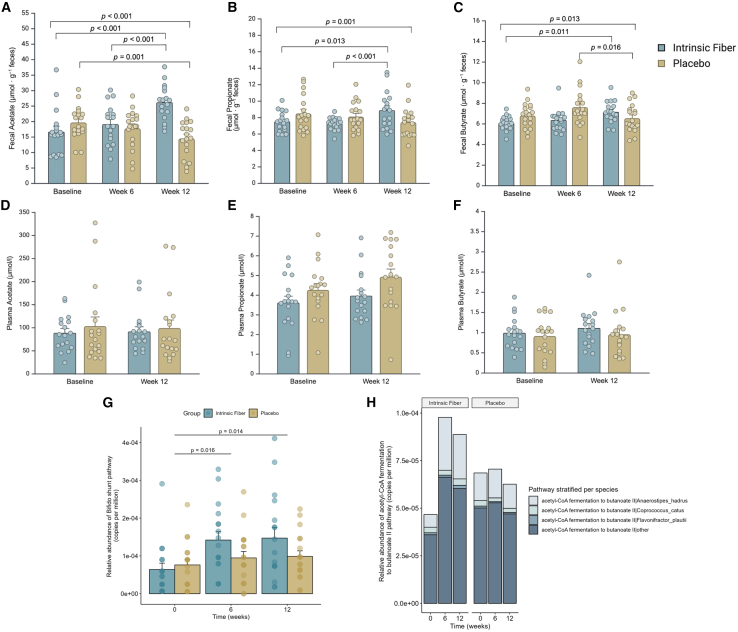
Change in fecal and plasma short-chain fatty acid concentrations, bifid-shunt, and butyrate production microbial pathways after 12-week of intrinsic fiber or placebo intervention Data are mean ± SEM for (A)–(F). (A) Fecal acetate, (B) fecal propionate, (C) fecal butyrate, (D) plasma acetate, (E) plasma propionate, and (F) plasma butyrate. Data were analyzed using linear-mixed model analysis with *p* values representing group × time interactions (*p* values above upper line). *Post hoc* analysis was done with a Fisher’s least significant difference test. (G) Relative abundance of the bifid-shunt pathway (copies per million) at baseline, week 6 and week 12 in the intrinsic fiber and placebo group. (H) Changes in the relative abundance (copies per million) of the butyrate production pathway based on functional profiling using metagenomics, notably attributed to increases in *Anaerostipes hadrus*, the most abundant *Anaerostipes* species. Created with BioRender.com.

The SCFAs acetate, propionate, and butyrate are the major end products of the microbial fermentation of dietary fibers. SCFA concentrations in fecal and plasma samples were assessed at multiple time points allowing for a comprehensive evaluation of gut microbial dynamics.^26^ No differences between groups were observed in fasting plasma acetate, propionate, and butyrate concentrations (Figures 3D–3F). Fecal acetate, propionate, and butyrate levels increased at week 12 by 56%, 18.2%, and 17.4%, respectively (*p* < 0.05 compared to placebo, Figures 3A–3C). The concentrations of acetate, propionate, and butyrate expressed as percentage of total SCFA were 55%:25%:20%, respectively, at baseline, and were 58%:22%:19% at week 6 and 62%:21%:17% after 12 weeks in the intrinsic fiber group, indicating a shift toward increased acetate production. The increase in fecal SCFA content in the intrinsic fiber group coincided with the increase in *Bifidobacterium* spp. and *Anaerostipes* spp. Both genera contain important SCFA-producing bacteria. Specifically, *Bifidobacterium* and *Anaerostipes* species have been shown *in vitro* to form butyrogenic trophic chains through the degradation of inulin by *Bifidobacterium* spp., with subsequent cross-feeding on produced lactate and acetate by *Anaerostipes* spp. leading to butyrate production.^27^^,^^28^ We previously confirmed that the formation of a butyrogenic trophic chain from dried chicory root was possible and needed the presence of a pectin-degrading *Bacteroides* strain, to open up the plant cells.^20^ Here, through metagenomic functional profiling, we confirm that both the unique degradation of sugars into lactate and acetate by *Bifidobacterium* spp, known as the bifid-shunt pathway (week 6: *p* = 0.016; week 12: *p* = 0.014), and the acetyl-coenzyme A (CoA) fermentation to butanoate II pathway (butyryl-Co:acetate CoA-transferase pathway; week 6: *p* < 0.001; week 12: *p* < 0.001) were significantly increased in the intrinsic fiber group compared to the placebo group (Figure 3G). Moreover, *Anaerostipes hadrus* had the largest contribution to the acetyl-CoA fermentation to butanoate II pathway (Figure 3H). These findings underscore our previous findings of a butyrogenic trophic chain being formed from the intrinsic fiber product *in vivo.* Changes in taxa at the genus level in the placebo group were not significant.

In summary, our findings indicate an intrinsic fiber-induced compositional and functional shift toward an increased abundance of genes related to saccharolytic fermentation in particular butyrate and propionate production pathways in the colon. Building on these findings, we investigated how changes in metabolic health markers, particularly those associated with T2D risk, could be influenced by increased fiber consumption, examining the potential metabolic health benefits linked to higher intrinsic fiber intake.

### Intrinsic fiber increases peripheral and whole-body but not hepatic and adipose insulin sensitivity

**Figure 4:**
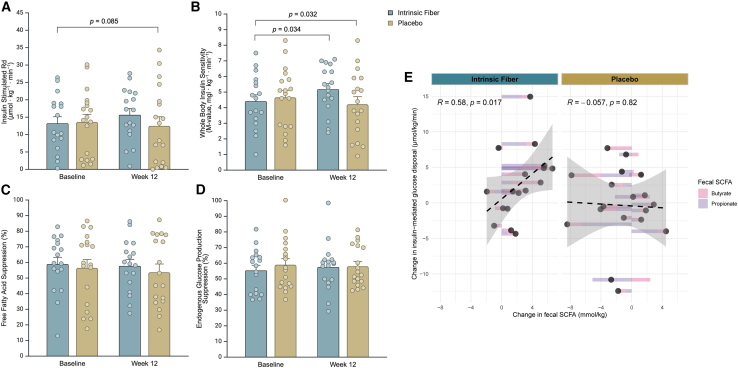
Change in peripheral, hepatic, and adipose tissue insulin sensitivity after 12-week intrinsic fiber (fiber) or placebo intervention (A) Insulin-stimulated glucose disposal (Rd). (B) Whole-body glucose disposal (M-value). (C) Adipose tissue insulin sensitivity assessed as insulin-mediated suppression (%) of circulating free fatty acids (FFAs). (D) Hepatic insulin sensitivity assessed as insulin-mediated EGP suppression (%). (E) Spearman correlation between change in fecal SCFA (butyrate and propionate) and the change in insulin-mediated glucose disposal in the intrinsic fiber group and in the control group. A stacked bar is indicated for the change in butyrate (pink) and propionate (purple). The points represent the average of these two changes. Individual correlations between propionate and butyrate and insulin-mediated glucose disposal are given in Figure S5. Data are mean ± SEM. Data for (A)–(D) were analyzed using linear-mixed model analysis with *p* values representing group × time interactions (*p* values above upper line). *Post hoc* analysis was done with a Fisher’s least significant difference test. Created with BioRender.com.

Our primary outcome measure was peripheral insulin sensitivity (insulin-stimulated glucose rate of disappearance, Rd) determined by the gold-standard two-step hyperinsulinemic-euglycemic clamp with [6,6 – ^2^H_2_]- glucose tracer infusion. Glucose concentrations as well as plasma ^2^H_2_-glucose enrichment reached a steady state during baseline and the last 30 min of the low and high step of the clamp. There was a tendency toward an increased peripheral insulin sensitivity in the intrinsic fiber group as compared to placebo (*p* = 0.085, Figure 4A), indicating an enhanced capability of peripheral tissue for glucose uptake. Non-oxidative glucose disposal was increased in the intrinsic fiber group as compared to placebo (*p* = 0.027, Table S11). Furthermore, there was an increase in whole-body insulin sensitivity (M-value) in the intrinsic fiber group compared to placebo (*p* = 0.032, Figure 4B). Interestingly, fecal butyrate and propionate concentrations changed in parallel to changes in peripheral insulin sensitivity, showing a positive correlation between the change in the sum of both SCFAs and insulin-mediated glucose disposal in the intrinsic fiber group and not in the placebo group (Figures 4E and S5).

Interestingly, in contrast to the trend toward an increased peripheral sensitivity and the increases in non-oxidative glucose disposal and whole-body insulin sensitivity, we observed no intrinsic fiber-induced change in adipose tissue (insulin-mediated free fatty acid [FFA] suppression, Figure 4C) or hepatic (suppression of endogenous glucose production [EGP], Figure 4D) insulin sensitivity. Data on glucose infusion rate, M-value, rate of glucose appearance, glucose disposal rate (rate of glucose disappearance), and EGP during the different steps of the 2-step hyperinsulinemic-euglycemic clamp are indicated in Table S11.

Recent studies have highlighted the critical role of the gut microbiota on tissue-specific insulin sensitivity. In a Swedish cohort including participants naive to diabetes treatment and grouped by glycemic status, the gut microbiota composition was altered in those with impaired glucose tolerance (IGT), and combined glucose intolerance and T2D, but not in those with impaired fasting glucose (IFG).^29^ Additionally, the abundance of several butyrate producers and the functional potential for butyrate production were decreased in both prediabetes and T2D groups.^29^ Importantly, IGT and IFG have previously been associated with peripheral and hepatic IR, respectively.^30^ Furthermore, modification of the gut microbiota, through either supplementation of dietary fiber or transplantation of fecal microbiota from lean insulin-sensitive individuals, has been shown to improve peripheral insulin sensitivity more effectively than hepatic insulin sensitivity in individuals with the metabolic impairments.^31^^,^^32^^,^^33^ Thus, in line with these studies, our findings suggest that microbial metabolism and the increased SCFA-producing pathways may be more strongly linked to peripheral non-oxidative glucose disposal and insulin sensitivity.

To further assess gut microbiota modulation of SCFA production on energy and substrate metabolism, we determined arterialized plasma metabolite concentrations and measured energy expenditure and substrate oxidation during the two-step hyperinsulinemic-euglycemic clamp by indirect calorimetry using an open-circuit ventilated hood system. Neither intrinsic fiber nor placebo significantly affected basal and steady-state energy expenditure, carbohydrate or fat oxidation, or metabolic flexibility (change in respiratory quotient between baseline and the insulin-stimulated state, Table S12). Post-treatment plasma glucose concentrations were reduced in the intrinsic fiber group as compared to placebo (*p* = 0.04, Table S13), which is in line with the observed improvement in whole-body and peripheral insulin sensitivity, as discussed earlier.

Fasting plasma insulin concentrations, Hemoglobin A1c (HbA1c) levels, HOMA-IR, and fasting plasma inflammatory cytokines (tumor necrosis factor alpha [TNF-α], interleukin [IL]-6, IL-8, IL-10, and interferon [IFN]γ), as well as fasting plasma GLP-1 and PYY concentrations, were not different between groups (Table S13). Even though we did not observe changes in fasting plasma GI hormone concentrations and inflammatory profile, some studies reported that increased (fiber-derived) colonic SCFA availability can impact the production and secretion of GLP-1^13^ or PYY^12^ and has anti-inflammatory properties,^34^ whereas others, in line with the current findings, did not report effects on fasting GI hormone concentrations or circulating inflammatory markers.^33^^,^^34^ Nevertheless, this discrepancy suggests a complex interplay between dietary fiber intake and gut microbial species capable of influencing metabolic regulation, potentially specific to fiber- and/or microbial/metabolic phenotype-related mechanisms.

### Intrinsic fiber decreased circulating TGs and tended to reduce intrahepatic fat accumulation

**Figure 5:**
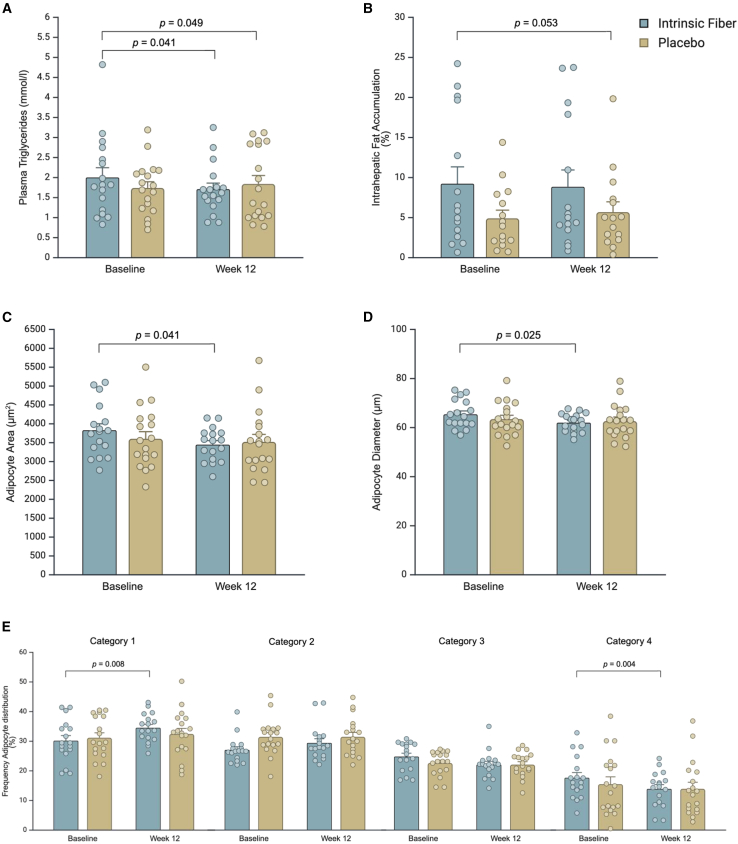
Change in plasma triglycerides, intrahepatic lipid accumulation, and adipocyte size frequency distribution 12 weeks after intrinsic fiber (fiber) or placebo intervention (A) Changes in fasting plasma triglycerides. (B) Changes in intrahepatic lipid expressed as percentage of total volume of liver. For (B), data are missing for *n* = 2 in fiber and *n* = 3 in placebo. (C) Adipocyte area (μM^2^). (D) Adipocyte diameter (μM). (E) Adipocyte size frequency was categorized into 4 distinct sizes: <50 μm, 50–69 μm, 70–80 μm, and >90 μm. Chi-squared analysis indicated a significant difference between the intervention groups (*p* < 0.001). *Post hoc* analysis conducted with pairwise comparisons using multiple z-tests of two proportions and Bonferroni correction with *p* < 0.0125 considered significant. Data presented as mean ± SEM. Data for (A) and (B) were analyzed using linear-mixed model analysis with *p* values representing group × time interactions. *Post hoc* analysis was done with a Fisher’s least significant difference test. *p* values in (C) and (D) indicate a significant time effect within the intrinsic fiber group. Created with BioRender.com.

Evidence from animal studies consistently demonstrates that increased dietary fiber-derived SCFA production is able to modify lipid metabolism and lower total cholesterol and TGs in the liver, ultimately improving liver health and reducing the risk of fatty liver disease.^35^ To address this, we assessed blood lipid spectrum including fasting TG, total cholesterol, and high-density lipoprotein (HDL) concentrations and assessed intrahepatic lipid (IHL) content by *in vivo* proton magnetic resonance spectroscopy (1H-MRS) performed on a 3-T magnetic resonance system using 32-channel sense cardiac/torso coil (Philips Healthcare). Our results did not indicate any differences in total cholesterol or HDL concentrations between groups. However, 12 weeks of intrinsic fiber intake resulted in significant reductions in circulating plasma TG (*p* = 0.049, Figure 5A) and a trend in decreasing IHL accumulation compared to placebo (*p* = 0.063, Figure 5B) after 12 weeks. This differentiates the efficacy of the intrinsic fiber product from a previous study, in which no changes in IHL accumulation were observed.^36^ These metabolic improvements observed in our study participants may, therefore, be partly attributed to the gut microbiota-facilitated increase in SCFAs or butyrate production. Indeed, we found a significant correlation between increases in fecal butyrate and improvements in plasma TG levels (r = −0.54, *p* = 0.024, Figure S4A). Butyrate has been shown to activate AMP-activated protein kinase (AMPK) and suppress Sterol regulatory element-binding protein-1 (SREBP-1c), thereby inhibiting lipogenic gene expression and hepatic *de novo* lipogenesis.^37^ Furthermore, propionate is described to initiate signaling pathways in hepatocytes, resulting in reduced gene and protein expression of lipogenic enzymes, leading to a reduced hepatic TG concentration.^38^ Mechanistic studies describe the ability of propionate to specifically increase the formation of odd-chain fatty acids, which correlate with the attenuation of high-fat diet-induced IR, emphasizing the role of propionate in improving insulin sensitivity and metabolic health.^38^ The fiber-induced increase of SCFAs reported earlier might suggest that the beneficial effects of intrinsic fibers on IHL and TG levels are related to these microbial metabolites. Indeed, several *in vivo* rodent studies with SCFA supplementation reduced hepatic fat accumulation and lipid metabolism with the underlying mechanisms hypothesized to increase hepatic lipid oxidation via an AMPK-acetyl-CoA carboxylase pathway, as well as reduced hepatic fatty acid synthase activity.^39^^,^^40^^,^^41^^,^^42^^,^^43^^,^^44^

### Intrinsic fibers increase the proportion of small adipocytes

Adipose tissue dysregulation is central to the pathophysiology of obesity and related metabolic issues.^45^ In our study, subcutaneous adipose tissue biopsies from abdominal regions provided insights into the effects of gut microbial fermentation of intrinsic fibers on physical characteristics of metabolic organs. While there were no significant differences between treatment groups, adipocyte diameter and adipocyte area decreased in the intrinsic fiber group (*p* < 0.05), while there was no change in the placebo group (Figures 5C and 5D). We subsequently categorized adipocyte sizes into four distinct groups with the counted adipocytes categorized into <50 μM, 50–69 μM, 70–80 μM, and >90 μM in diameter. Our results indicate an increase in adipocyte sizes <50 μm (from 30.2% pre-intervention to 34.5% post-intervention, *p* = 0.008) and a decrease in adipocyte sizes >90 μm (from 17.4% pre-intervention to 13.9% post-intervention, *p* = 0.004, Figure 5E) in the intrinsic fiber group. There were no significant changes in any adipocyte size categories after 12 weeks in the placebo group. These findings demonstrate a re-distribution of adipocyte size in response to intrinsic fiber supplementation independent of changes in body weight. Interestingly, in our study, increases in fecal butyrate tended to be associated with increases in small adipocytes (r = 0.45, *p* = 0.073, Figure S4B) in the intrinsic fiber group. Therefore, modulating colonic and circulating SCFAs may be crucial for regulating human adipose tissue lipid metabolism. These findings highlight the role of SCFAs as key mediators of adipocyte function, potentially leading to changes in cell morphology and function.

### Intervention response is driven by increases in gut microbial butyrate pathways and circulating acetate concentrations

After intervention, a trend toward an increase in peripheral insulin sensitivity, our primary outcome, was observed within the intrinsic fiber group with a high interpersonal variability (Figure S6). Furthermore, the response of the microbiota to dietary stimuli and the conversion of dietary fiber into SCFAs by the gut microbiota are highly individualized processes, influenced by an individual’s gut microbiota signatures, and may therefore determine metabolic outcome response.^46^^,^^47^ To further explore this, participants were categorized into two subgroups based on a cutoff of a clinically relevant 15% increase in peripheral insulin sensitivity. Those experiencing more than a 15% improvement were classified as high responders (*n* = 9), and those with 15% or less as low responders (*n* = 8, Figure S6). The high responders were slightly younger, and the observed richness of the microbiota was slightly higher, with no differences in other baseline characteristics between groups (Table S14).

**Figure 6:**
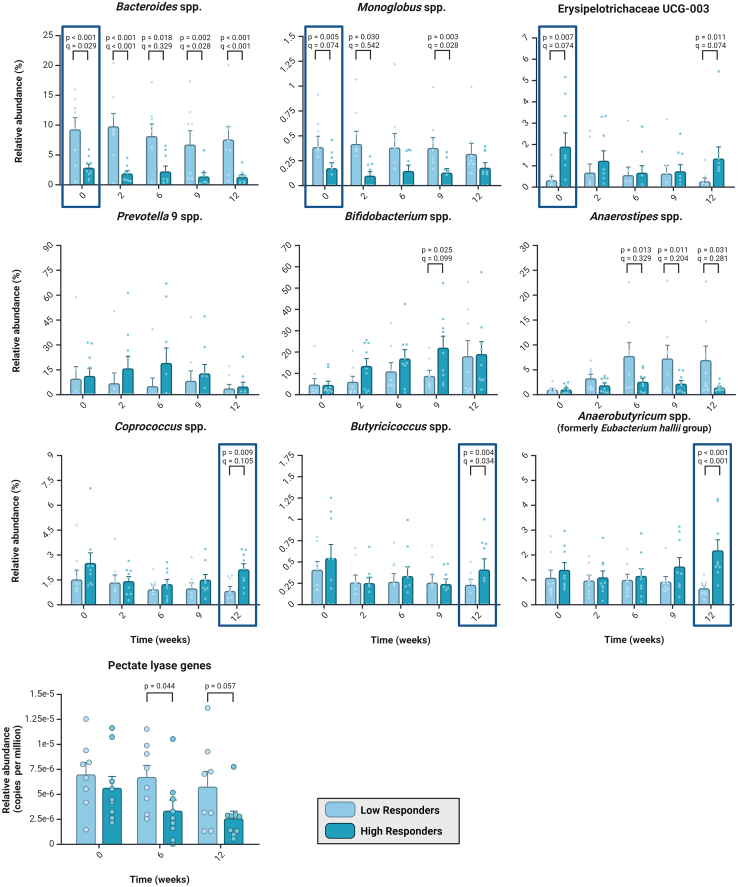
Gut microbiota differences between low responders and high responders at baseline (week 0) and over the course of the intervention (week 2, 6, 9, and 12) Differential abundance testing with false discovery rate (FDR) correction (*q* values) was performed as implemented in mare (see statistical methods). Created with BioRender.com. Created with BioRender.com.

With respect to shifts in the fecal microbiota composition, high responders had significantly lower relative levels of *Bacteroides* spp. and *Monoglobus* spp. and significantly higher levels of *Erysipelotrichaceae* UCG-003 spp. compared to low responders (Figure 6). These differences persisted throughout the intervention to week 12, although their relative levels decreased over time. Metagenomic profiling revealed that the most abundant *Bacteroides* species included *B. uniformis* and *B. xylanisolvens*, both known pectin degraders,^48^ and that *Monoglobus pectinilyticus* was detected at the species level, which is also a dedicated pectin degrader.^49^ Pectin degradation by these species is known to result in the formation of propionate.^50^ The observation of lower relative levels of pectin-degrading taxa was in line with the lower relative abundance of pectate-lyase-encoding genes in high responders compared to low responders, notably at weeks 6 and 12 (Figure S7). Aside from these baseline differences, we observed that relative abundances of *Prevotella* 9 spp. (primarily composed of *Segatella* spp.) and *Bifidobacterium* spp. increased rapidly (at week 6) in the high responders, with the latter mainly attributed to higher abundances of *Bifidobacterium adolescentis* at the species level (Figure S8). In contrast, relative abundances of *Anaerostipes* spp. increased more rapidly in low responders. Finally, several butyrate and propionate producers, including *Butyricicoccus* spp., *Coprococcus* spp., and the *Anaerobutyricum* spp., formerly known as *Eubacterium hallii* group,^51^ reached significantly higher final relative abundances in high responders compared to low responders (Figure 6; Figure S8). Of note, the phylogenetically related bacteria *Anaerostipes* spp. and *Anaerobutyricum* spp. have the unique capacity to convert lactate and acetate into butyrate through a metabolic pathway highly conserved among butyrate producers.^52^

Next, we investigated how these differences in gut microbial profiles in low responders and high responders were reflected in fecal SCFAs, plasma SCFAs, and other metabolic health measures. Our results demonstrate that the high responders had the greatest increase in fecal butyrate and propionate concentrations (*p* = 0.0063 and *p* = 0.031, respectively. Table S15). In addition to the changes in SCFAs, we observed marked differences in systemic metabolic health markers. High responders showed a large decrease in TG levels (*p* = 0.046) and an increase in HDL levels (*p* = 0.019) as well as in small adipocytes (*p* = 0.033, Table S15).

In addition to the aforementioned relation between increased fecal butyrate and decreased circulating TG and an increased percentage of small adipocytes, we found that higher increases in fecal butyrate were related to higher increases in plasma acetate levels (Figure 4D). Of interest, high responders showed a greater percentage increase in plasma acetate concentrations, with a relative increase (% change) of 36.7% in high responders compared to −10.9% in low responders (% change, *p* = 0.045, Table S14). The benefits of acetate on metabolic health outcomes, particularly through modulating adipose tissue function, have been demonstrated before. Cell line and animal models have shown that acetate may improve hepatic glucose and lipid metabolism, as well as adipose lipid metabolism, potentially through G protein-coupled receptor 43 (GPR43).^3^^,^^34^ In humans, distal infusions of SCFA, in particular acetate, have been shown to reduce whole-body lipolysis, and related changes in plasma acetate levels were linked to higher fat oxidation and resting energy expenditure.^12^ Of note, circulating acetate appeared to correlate inversely with the percentage of large adipocytes in the intrinsic fiber group and not in the control group (Figure S4F).

Overall, interindividual variability in response to intrinsic fiber intake was prominent with some participants increasing peripheral insulin sensitivity by up to 3-fold compared to baseline, while in others values remained unchanged. Changes in peripheral insulin sensitivity occurred independent of changes in body weight, activity level, and dietary intake. Our data indicate that changes in the gut microbiota, and the related increased butyrate and propionate production and systemic acetate levels, are an important mediator of this variability in the improvement of metabolic parameters.

## Discussion

Epidemiological evidence supports a pronounced role of dietary fiber in the prevention of non-communicable disease.^22^ These results are seldom confirmed in studies with isolated fiber extracts since fiber within an intact plant matrix structure may be of importance for a more gradual plant cell fiber release and more distal colonic fermentation and SCFA production, in particular butyrate, which may be crucial for cardiometabolic health effects. In this randomized, double-blind, placebo-controlled trial, we examined the effects of a 12-week supplementation with a minimally processed intrinsic dietary fiber from dried chicory root cubes in adults with overweight/obesity at risk of T2D. The addition of the intrinsic fiber product led to a trend toward improved peripheral insulin sensitivity and significant increases in non-oxidative glucose disposal and whole-body insulin sensitivity, reduced circulating TG levels, a trend toward decreased IHL content, and a reduced adipocyte size compared to placebo. Fecal microbiota analyses indicated that these positive metabolic health effects were paralleled by substantial increases in the relative abundance of *Bifidobacterium* spp. and *Anaerostipes* spp. that have the capacity to form a butyrate-producing trophic chain.^23^^,^^27^ These findings highlight the potential of intrinsic fibers from dried chicory root cubes in modulating gut microbiota composition and improving metabolic health.

In our study, intrinsic fiber intake resulted in a prominent shift in the gut microbiota composition and fecal SCFA profiles compared to placebo, with a specific enrichment of butyrate-producing taxa and a temporal increase in fecal butyrate, propionate, and acetate concentrations throughout the study period. Our 16S rRNA gene sequencing data show a 4- to 5-fold increase in *Bifidobacterium* spp. and *Anaerostipes* spp. relative abundances at genus level. Hence, although taxonomy-based summary measures like β-diversity were different between groups up to 9 weeks, this was not significant anymore at week 12, while specific microbial taxa still appeared to change. Consequently, we do not consider the absence of statistically significant differences in β-diversity as indicative of the efficacy of the intervention. Taxonomic profiling based on metagenome sequencing confirmed the presence of the species *Bifidobacterium longum* alongside *Bifidobacterium bifidum* and *Bifidobacterium adolescentis* as well as the exclusive presence of *Anaerostipes hadrus.* Furthermore, high relative abundances of known butyrate producers and propionate producers were observed with intrinsic fiber intake, notably in high responders. Additionally, in high responders, we observed low relative abundances of pectin-degrading bacteria and pectate-lyase-encoding genes. Pectin degradation results in the opening of plant cells, which allows for the release of fiber and the access of intracellular inulin, thereby facilitating saccharolytic fermentation and the formation of a butyrogenic trophic chain. Consequently, the low levels of pectin-degrading bacteria and genes in high responders (and vice versa) suggest a slow opening of the plant cell wall, resulting in a subsequent distal colonic release of fiber from the plant cells that are fermented there into mainly butyrate and propionate.^50^ In an earlier study, it was found that, in individuals with obesity, low fecal pectate lyase protein levels correlated with low IR.^53^ Our findings indicate that the highest improvements in insulin sensitivity were observed in individuals where the intrinsic fiber plant cells possibly opened slowly. This suggests a potential mechanism through which intrinsic fiber may have modulated the gut ecosystem to support enhanced butyrate and propionate production in the distal colon. This modulation likely occurred by promoting a lower intestinal pH and the preferential growth of butyrate- and propionate-producing taxa. This is supported by findings from *in vitro* fecal batch fermentations, showing that butyrate production from dried chicory root occurred at a late stage during fermentation, which, *in vivo*, would translate into a distal butyrate production.^20^

In the present randomized clinical trial, the evident changes in microbial species involved in butyrate and propionate production as well as fecal SCFA, butyrate, and propionate concentrations with the intrinsic fiber dried chicory root product led to a trend toward enhanced peripheral insulin sensitivity and a significantly increased non-oxidative glucose disposal and whole-body insulin sensitivity in individuals with overweight or obesity at risk for T2D. This was accompanied by decreased circulating TG concentrations, a tendency toward a reduced IHL content, and a shift in adipocyte cell size toward a higher frequency of small adipocytes. Human studies that focus on the impact of soluble, isolated fibers on parameters of glucose and lipid homeostasis, in particular in people at risk of T2D, are controversial. A previous study from our group noted a strong increase in *Bifidobacterium* spp. abundance after 12-week supplementation of 15 g galacto-oligosaccharides daily, rapidly fermented in the proximal colon, while no increase in fecal or plasma SCFAs or improved metabolic outcomes were observed.^54^ Other studies utilizing isolated fibers or fiber derivatives also reported discrepancies between the presence of SCFA-producing bacteria and actual SCFA levels or metabolic improvements.^55^^,^^56^^,^^57^^,^^58^^,^^59^^,^^60^^,^^61^ To elucidate the role of site of fermentation or administration of SCFA in the controversial results, we previously demonstrated that direct SCFA infusion into the distal colon showed better metabolic outcomes than proximal administration, suggesting that the site of fiber fermentation significantly affects its efficacy.^12^ Based on that, we hypothesized that particularly fibers or fiber mixtures leading to distal saccharolytic fermentation would lead to significant health benefits. Our current findings highlight the importance of utilizing a minimally processed, intrinsic fiber source. We propose that this leads to a slower release of fiber in the colon,^20^ thereby prolonging saccharolytic fermentation toward the distal colon and prominently improving cardiometabolic health.

The improvement in whole-body insulin sensitivity in the present study was mainly attributed to a trend toward increased peripheral insulin sensitivity and increased non-oxidative glucose disposal with no effects on hepatic insulin sensitivity, suggesting that gut microbial modulation of insulin sensitivity may have a tissue-specific nature. This is in line with improvements in peripheral rather than hepatic insulin sensitivity that were reported following both dietary maize fiber intervention and fecal microbiota transfer in people at cardiometabolic risk.^31^^,^^32^^,^^33^ As mentioned earlier, in our previous research, we found that distal colonic SCFA infusion and not proximal infusion went along with marked increases in systemic SCFA and in energy expenditure and fat oxidation, which were correlated with the systemic circulating SCFAs and in particular acetate concentrations.^12^ These data may suggest that the systemic increase in circulating acetate is required to induce these effects. Interestingly, we previously showed that the distal colon releases a 2-fold higher amount of SCFA as compared to the proximal colon *in vivo* in men.^62^ In the present study, no significant increases in plasma SCFA were found. Nevertheless, when dividing the group into high responders and low responders based on percentage increase in peripheral insulin sensitivity, the percentage increase in circulating acetate was significantly higher in the high responder as compared to the low responder group. The latter finding along with more distinct changes in circulating TG, adipocyte size, and HDL cholesterol and an inverse correlation of change in circulating acetate and the percentage large adipocytes suggests a putative role of circulating acetate in modulating peripheral insulin sensitivity via several mechanisms. Besides a role of systemic acetate as a mediator for the effects on peripheral insulin sensitivity, other SCFA-related mechanisms may also be involved. In addition to the increased presence of butyrate- and propionate-producing species, fecal butyrate and propionate levels were elevated. These SCFAs may act as ligands for the receptors GPR41 and GPR43 present in the gut and in all metabolically active tissues, inhibit cyclic AMP accumulation, and activate mitogen-activated protein kinase signaling, thereby enhancing glucose and lipid oxidation and energy expenditure. Notably, based on circulating butyrate concentrations and the IC50 for butyrate, direct peripheral effects of butyrate through GPR receptors may be unlikely. Nevertheless, an effect cannot be excluded since tissue extracellular butyrate concentration may be considerably higher than that in the systemic circulation. Thus, effects of butyrate and propionate on peripheral insulin sensitivity may be mediated by gut signaling via GPR or possibly via direct peripheral effects, as evidenced by increased fecal butyrate and propionate concentrations in high responders to the intervention, most likely reflecting increased SCFA production toward the distal colon. This is supported by the direct correlation between the sum of the change in fecal butyrate and propionate and peripheral insulin sensitivity. Besides that, the effect may also be indirect through effects on liver or adipose tissue metabolism. Indeed, there was a strong tendency toward reduced IHL content and a significantly reduced circulating TG concentration, most likely reflecting liver very low density lipoproteins (VLDL)-TG output in the intrinsic fiber group as compared to placebo. Moreover, fecal butyrate was significantly associated with circulating TG concentrations. Effects on IHL content and systemic TG may be mediated by effects of butyrate on liver metabolism through effects on AMPK activity, thereby affecting fat oxidation and the balance between oxidation, storage, and release. As a consequence, liver TG output and circulating concentrations are impacted, which may in turn affect peripheral insulin sensitivity.^63^

As indicated earlier, an important determinant of the beneficial metabolic effects may be a modulation of adipose tissue metabolism. Indeed, we observed a decrease in adipocyte area and a shift toward smaller adipocytes in the intrinsic fiber group as compared to placebo. The exact mechanism for this positive effect on adipocyte size remains to be determined. Fecal butyrate concentrations, as well as circulating acetate concentrations, were associated with a reduced adipocyte size. The benefits of acetate on modulating adipose tissue function have been demonstrated before. Cell line and animal models have shown that acetate may improve hepatic glucose and lipid metabolism, as well as adipose lipid metabolism, potentially through G protein coupled receptor (GPR) 43.^3^ In humans, distal infusions of SCFAs have been shown to reduce whole-body lipolysis, and related changes in plasma acetate levels were linked to higher fat oxidation and resting energy expenditure.^12^ In line, a study using maize starch showed increased systemic acetate and propionate concentrations together with effects on adipose tissue metabolism, including an increased expression of lipolytic enzymes reflecting an increased adipocyte differentiation.^33^ In summary, the intrinsic fiber dried chicory root product resulted in higher colonic SCFA content and systemic acetate concentrations, which in turn reduced IHL content, improved blood lipid profile, and reduced adipocyte size and peripheral insulin sensitivity in individuals with obesity.

Several strengths of our study include the robust double-blinded randomized, placebo-controlled design, use of gold-standard methods, extensive metabolic phenotyping, detailed assessment of gut microbiota changes, and examination of relevant mechanistic pathways. The current study offers a comprehensive exploration of the metabolic and gut microbiota effects of an intrinsic dietary fiber intervention compared to placebo in an at-risk population for T2D.

In conclusion, 12 weeks of intrinsic fiber intake modifies gut microbial metabolism and improves metabolic health putatively through an increased abundance of butyrogenic bacteria, which are adjacent to cross-feeding networks, and enhances butyrate production. Additionally, in the high responders to the intrinsic fiber intake, microbial propionate producers as well as fecal propionate were increased, likely due to the degradation of pectin that is present in the plant cell walls of the dried chicory root. Our findings demonstrate an important role of intrinsic dried chicory root fibers in modulating microbial SCFA-producing pathways and fecal SCFAs, as well as whole-body and peripheral insulin sensitivity, IHL content, and adipocyte cell size. These findings support incorporating intrinsic fibers into long-term dietary strategies as a low-risk approach to manage and improve metabolic health. We anticipate that these results offer an additional perspective on dietary fiber interventions and the importance of the intrinsic dietary fiber structure as a preventive strategy against T2D and cardiometabolic disease.

### Limitations of the study

There are several limitations of the present study. At first, although several measures of insulin sensitivity including whole-body insulin sensitivity and non-oxidative glucose disposal were increased as a result of intrinsic fiber supplementation, our primary outcome only showed a trend toward significance (*p* = 0.085). Furthermore, direct evidence for causality between distal colonic SCFA and colonic SCFA and butyrate production and the beneficial metabolic effects is supported by our previous research^11^^,^^12^ but is hard to provide in the current randomized controlled trial. Thirdly, we measured plasma SCFA and metabolic satiety indicators like PYY and GLP-1 only in a fasted state, which does not reflect their postprandial variations and narrows our understanding of their dynamics. In addition, due the absence of a meal test, we could not evaluate cardiometabolic responses to food intake, limiting insights into how intrinsic fibers influence key metabolic health markers such as insulin and glucose regulation. Lastly, the duration of our study was 3 months, and it would be of special interest to study benefits in the long term.

## Resource availability

### Lead contact

Further information and requests for resources and reagents should be directed to and will be fulfilled by the lead contact, Ellen E. Blaak (e.blaak@maastrichtuniversity.nl).

### Materials availability

This study did not generate new unique reagents.

### Data and code availability

16S rRNA gene amplicon and shotgun metagenomic sequences have been submitted to the European Nucleotide Archive under the accession number PRJEB81868. Code is accessible via https://git.wur.nl/marie-luise.puhlmann/omary-et-al-2025/ (digital object identifier: https://doi.org/10.5281/zenodo.15532890).

Any additional information required to reanalyze the data reported in this paper is available from the lead contact upon request.

## Acknowledgments

We would like to sincerely thank our study participants for participating in the study. We thank W. Sluijsmans, H. Aydeniz, F. Bouwmans, G. Hul, and N. Hoebers for their excellent support with performing lab analyses. We thank P. Roosendaal for her indispensable help with the fecal DNA extraction and 16S rRNA gene PCRs, as well as A. Jonkers, I. Heikamp-de Jong, and M. van Gaal from the Laboratory of Microbiology, Wageningen University & Research. Funding for this research was obtained from the EFSD/Lilly European Diabetes Research Program 2019 as well as Topconsortia voor Kennis en Innovatie (TKI)/Health Holland (LSHM19050). We thank WholeFiber Holding BV for kindly providing us the chicory-derived intrinsic fiber product. The graphical abstract was created with BioRender.com.

## Author contributions

L.O. and M.-L.P. conducted the research, acquired data, and completed statistical analysis. L.O., E.E.C., and M.-L.P. drafted the manuscript. A.G., J.J.H., and Y.M.H.O.d.K.-B. generated data. E.E.C., W.M.d.V., and E.E.B. obtained funding for this project. E.E.B. was the project leader and principal investigator. All authors actively participated in discussion of results, and revision of the article, and approved the final version of the manuscript.

## Declaration of interests

I.R. is employed by and W.M.d.V. is a scientific board member of WholeFiber Holding BV, which provided the study material.

## STAR★Methods

### Key resources table

**Table:** 

REAGENT or RESOURCE	SOURCE	IDENTIFIER
**Chemicals, peptides, and recombinant proteins**		

Stool Transport and Recovery (STAR) buffer	Roche Diagnostics	Cat# 03335208001
Nuclease free water	Qiagen	Cat# 129114
Phusion Green HF buffer	ThermoFisher	Cat# F538L
dNTPMix	Promega	Cat# U1515
Phusion Hot start II DNA 2 U/μL polymerase	ThermoFisher	Cat# F549S
2.2% Aragose Gel	Lonza	Cat# 57032

**Critical commercial assays**		

Maxwell® 16 Tissue LEV Total RNA purification Kit	Promega	Cat # XAS1220
Qubit™ dsDNA Quantification BR Assay Kit	ThermoFisher	Cat# Q32850
CleanPCR kit	CleanNA	Cat# CPCR-0500
Human Insulin Kit (Elisa)	Crystal Chem (Europe), Zaandam	Cat.# 90095
Glucose HK CP ABX Pentra	Horiba ABX	Cat# A11A01667
FFA standard enzymatic assay on ABX Pentra	WAKO Instruchemie, The Netherlands	Cat. R1 #91696/Cat. R2 #91898 Cat. Standard #91096
Inflammatory markers	Meso Scale Discovery, Rockville, Maryland	Cat. #K151A9H-1

**Deposited data**		

V4–16S rRNA amplicon sequences and shotgut metagenome sequences	European Nucleotide Archive	Primary accession PRJEB81868. Code is accessible: https://git.wur.nl/marie-luise.puhlmann/omary-et-al-2025/ Digital Object Identifier: https://doi.org/10.5281/zenodo.15532890;

**Oligonucleotides**		

Primer: 515F (5′-GTGYCAGCMGCCGCGGTAA-3′)	(Parada et al.^64^)	N/A
Primer: 806R (5′-GGACTACNVGGGTWTCTAAT-3′)	(Walters et al.^65^)	N/A

**Software and algorithms**		

R version 4.2.3 R packages mare as well as the vegan, phyloseq, microbiome and microViz	R Core Team	https://www.r-project.org/
Linear Mixed Model Analysis and Chi-squared analysis using IBM SPSS v28.	IBM	https://www.ibm.com/products/spss-statistics

**Other**		

Dried chicory root particles	WholeFiber Holding BV	www.wholefiber.nl
Puffed Millet	Biovoordeel.nl	Article number: 300012170

### Experimental model and study participant details

#### Study participants

Forty-two men and women aged 45–70 years with overweight/obesity (BMI ≥28 kg/m2 < 35 kg/m2), who as well as had either insulin resistance (Homeostatic model assessment for insulin resistance value (HOMA-IR) > 2.2), impaired fasting glucose (5.6 < 7 mmol/L) and/or impaired glucose tolerance (7 < 11 mmol/L 2-h post OGTT)^24^ were recruited between January 2021 and January 2023 via a volunteer database, flyers and advertisements in local and online media. Further inclusion criteria included self-reported body weight stability for at least 3 months (no gain or loss >3kg), normal blood pressure range (systolic blood pressure 100–140 mmHg, diastolic blood pressure 60–90 mmHg). Exclusion criteria included participants who had been diagnosed with Type 2 Diabetes mellitus (defined as fasting glucose >7 mmol/L and 2h glucose >11 mmol/L), gastrointestinal diseases or had undergone abdominal surgery; diagnosed with cardiovascular diseases, cancer, liver or kidney malfunction. In addition, substance abuse, excessive smoking (defined as >20 cigarettes per day), plan to lose weight or follow a diet, supplementation with prebiotics or probiotics on a regular basis, exercise intensively for more than 3 h a week, medication used that influences glucose or fat metabolism and inflammation (NSAIDS), laxation use, prescribed antibiotic use within 3 months of starting the intervention, metal implants or tattoos on the head, shoulders, breast and neck or experiencing claustrophobia led to exclusion from the study. This human study was approved by the Medical Ethical Committee of Maastricht University Medical Center+ and was conducted in accordance with the Declaration of Helsinki (revised version, October 2008, Seoul, South Korea) and was registered at ClinicalTrials.gov (**NCT04714944**). Monitoring was performed by the independent Clinical Trial Center Maastricht (CTCM), Maastricht, The Netherlands. Written informed consent was obtained from all volunteers. All authors had access to the data of the clinical trial and reviewed the final version of the manuscript.

#### Assessment of eligibility

During the screening visit at the university, after an overnight fast (<10 h), standard anthropometric measurements were conducted for assessing body weight, height, waist-to-hip ratio, blood pressure (diastolic and systolic), fasting blood sampling for the determination of plasma glucose and insulin concentration, alanine aminotransferase, creatinine, HbA1C. Additionally, a 2-h oral glucose tolerance test was conducted for assessment of plasma glucose and insulin dynamics post-prandially. Participants ingested 200 mL of a ready-to-use 75 g glucose solution (Novolab, Geraardsbergen, Belgium) within 5 min, and blood samples were collected from the antecubital vein via an intravenous cannula under fasting conditions (t = 0) and after ingestion of the glucose drink (t = 30, 60, 90, and 120 min) for determination of plasma glucose and insulin concentrations. HOMA-IR was calculated based on fasting glucose and insulin measurements derived from the product of the insulin and glucose values divided by a constant, that is, calculated by using the following formula: fasting glucose (mmol/L) X fasting insulin (mU/L)/22.5.^66^

#### Study design and randomization

This randomized clinical trial was a placebo controlled, double-blind, randomized parallel design in 42 adult volunteers (Figure S2). After stratification for gender and age, an independent researcher randomized the participants into the intrinsic fiber or placebo group. The intervention period lasted 12 weeks (84 days) with a maximum of 90 days intervention. Both the participants and investigators were blinded to the treatment. All participants were instructed to continue their usual physical activity and dietary regimen throughout the whole intervention period.

The primary outcome of the study was the effect of the intrinsic fiber product on peripheral insulin sensitivity as measured by the Hyperinsulinemic-euglycemic clamp method. Secondary outcomes were hepatic and adipose insulin sensitivity, fecal microbiota composition and functionality, substrate oxidation and energy expenditure, body weight and composition, intrahepatic lipid accumulation, circulating metabolites (glucose, insulin, free-fatty acids, total cholesterol, triglycerides, high-density lipoproteins, low-density lipoproteins, TNF-α, IL-6, IL-8, IL-10, IFN-γ). Participants were also monitored for any changes in gastrointestinal symptoms through a gastrointestinal symptom questionnaire (GISQ). A schematic diagram outlining the study trajectory is displayed in Figure S1.

#### Study product and supplementation scheme

Forty-two participants were included and randomized into either treatment with a fiber product derived from chicory root (provided by WholeFiber BV, The Netherlands), or an iso-caloric placebo alternative from puffed millet (provided by DO-IT BV, The Netherlands). The dried chicory root product consists of chicory roots that have been washed, cut and dried, and has a dry weight of 93% (w/w), including 85% (w/w) fiber, whereof 70% (w/w) is native inulin, 10% (w/w) pectin and 5% (w/w) hemicellulose and cellulose. Furthermore, the dried chicory root product contained a variety of ‘non dietary fiber’ macro- and micro-nutrients, 15% of the solids of dried chicory root: protein (5%), minerals (K, Ca, P etc) + organic acids (together 5%), mono-/disaccharides (3%), polyphenols/vitamins (∼0.5%), fat (<1%).

During the intervention period, participants were instructed to consume either product divided over two equal daily dosages, one in the morning with breakfast and the second in the evening with dinner, for 12 weeks. For the first two-weeks, participants were given half the intended dose to help slowly acclimatize participants to the extra fiber intake. For the following 10 weeks participants consumed the full 30 g/day of intrinsic fiber or an iso-caloric amount (15.8 g/day) of placebo in the same manner. To check compliance, product intake was recorded in a diary format whereby participants indicated whether they have ingested the product with space for remarks in case the required intake for the day was not met. In addition, participants were required to keep the packaging in which the product was provided for a final count at the end of the study.

### Method details

#### Dietary intake and physical activity recording

Dietary intake was assessed by means of weighed 3-day food records before the start of the study, at week 6 and at the end of the study (week 12) intervention. Before the start of the study all participants were instructed on how to weigh and record their food and beverage intake. Participants were instructed to fill in their intake records on two weekdays and one weekend day. The dietary records were checked, discussed in case of missing data, and analyzed by the clinical investigator. Energy and nutrient intake were analyzed using the Dutch Food Composition Dataset (Nederlands Voedingsstoffenbestand, National Institute for Public Health and Environment, Ministry of Health, Welfare and Sport, The Hague, The Netherlands).

Additionally, self-reported levels of physical activity were assessed using the Short Questionnaire to Assess Health enhancing physical activity (SQUASH) before the start of the intervention, at week 6, and at the end of intervention (week 12). Before the start of the study, participants were instructed on how to record their physical activity. Outcome value was the time spent (minutes) in light, moderate, and vigorous physical activity based on metabolic equivalent, as reported before.^67^

#### Two-step hyperinsulinemic–euglycemic clamp

A two-step Hyperinsulinemic–euglycemic clamp combined with a [6,6-^2^H_2_]- glucose tracer (Cambridge Isotope Laboratories) was performed to measure the rate of glucose disosal (Rd), endogenous glucose production (EGP)^68^ and the insulin-mediated suppression of FFA.^68^ At t = −120 min, primed D-[6,6-^2^H_2_] glucose tracer was started and infused continuously at 0.04 mg/kg.min^−1^, to allow calculations of rates of EGP, glucose appearance (Ra), and the rate of glucose disposal (Rd) at basal conditions.^69^ Blood samples were taken from a superficial dorsal hand vein, which was arterialized by using a hot box (50^o^C). After a bolus-injection (2.4 mg/kg), tracer-infusion was started at 0.04 mg/kg/min, which was continued throughout the measurement. After 2 h, low-dose insulin was infused at 10 mU/m2/min^−1^ for 2 h,^70^ followed by high-dose insulin at 40 mU/m^2^/min^−1^ for 2 h^71^ By variable co-infusion of a 20% glucose solution, enriched by 1% tracer, plasma glucose concentrations were maintained at 5.0 mmol/L. For calculation of steady-state kinetics, additional blood samples were taken in the last 30 min of each step (0, 10, and 40 mU/m^2^/min insulin). Substrate utilization was measured for 30 min during the basal, low insulin, and high insulin infusion using indirect calorimetry by ventilated hood (Omnical, Maastricht Instruments, Maastricht). The clamp was performed after an overnight (≥12 h) fast and participants consume the standardized meal (Maaltijdpannetje Malse Kip, Aviko B.V, The Netherlands) the evening before the clamp.

#### Indirect calorimetry

Indirect calorimetry was used to assess the degree of substrate oxidation and energy expenditure during the steady state measurements of the two-step clamp. The open-circuit ventilated hood system was used (Omnical, Maastricht University).^72^ CO_2_ production (carbon dioxide output per unit of time in L/min) and O_2_ consumption (oxygen consumption per unit of time in L/min) were measured during three investigational time periods: a baseline measurement of 30 min before start of the clamp and for 30 min during the two steady-state of the Hyperinsulinemic–euglycemic clamp. Calculations of energy expenditure and substrate oxidation were performed according to the formulas of Weir^73^ and Frayn.^74^ Nitrogen excretion assumed that protein oxidation represents ∼15% of total energy expenditure.

#### Biochemical analyses for plasma variables

Venous blood was collected in EDTA tubes (Becton Dickinson, Eysins, Switzerland), which were centrifuged at 3000 g, 4°C for 10 min and plasma was aliquoted and directly snap-frozen in liquid nitrogen and stored at - 80°C until analysis of insulin, glucose, FFA and inflammatory markers. Plasma glucose was analyzed using a commercially available kit (ref. no. A11A01667, Glucose HK CP, ABX Diagnostics, Montpellier, France; and ref. no. HI-14K, Millipore, St. Louis, MO, respectively). Plasma FFA was analyzed using a commercially available kit (Ref. no reagent set 1 434–91795, Ref. no reagent set 2: 436–91995, ref. no. standards: 270–77000, Instruchemie, Zwet 26, 9932AB Delfzijl, The Netherlands) with the Cobas Pentra C400. Plasma triglycerides, total cholesterol and high-density lipoprotien (HDL) were measured using standard kits (Roche Diagnostics, Rotkreuz, Switzerland) on the Cobas Pentra C400. Plasma insulin was assessed using a Crystal Chem Human ELISA kit (Crystal Chem (Europe); Daalderweg 1, 1507DS, Postbus 9, 1500EA, Zaandam, The Netherlands). A 2-mL EDTA tube containing 20 μL of dipeptidyl peptidase-IV inhibitor (Millipore, Darmstadt, Germany) was sampled for GLP-1 analysis. Plasma samples were assayed for total GLP-1 immunoreactivity using an antiserum, which reacts equally with intact GLP-1 and the primary (N-terminally truncated) metabolite as previously described.^75^ A 2-mL aprotinin tube containing 20 μL of dipeptidyl peptidase-IV inhibitor was used to sample blood for plasma PYY analysis. Total PYY was assessed as described previously^76^ using a monoclonal antibody MAB8500 (Abnova, clone RPY-B12), which reacts equally well with PYY1 − 36 and PYY3 − 36. For plasma SCFA, blood was collected in a 4-mL Lithium Heparin tube (BD, Plymouth, UK) and analyzed only during fasted conditions prior to the start of the clamp procedure. Plasma acetate, propionate, and butyrate concentrations were determined using liquid chromatography–mass spectrometry (LC-MS) as previously described.^77^ Inflammatory cytokines were assessed using a Meso Scale Discovery Kit (Meso Scale Discovery, Meso Scale Diagnostics, USA). TNF-a, IFN-g, IL-6, IL-8 and IL-10 samples are added with a 60 μL solution containing detection antibodies conjugated with electrochemiluminescent (ECL) labels (MSD SULFO-TAG) over the course of one or more incubation periods.^78^

#### Analysis of fecal microbiota and SCFA concentrations

Fecal samples were collected at home at baseline, week 2, week 6, week 9 and week 12 of the intervention using feco-container buckets and divided into 3 sterile tubes. Participants were instructed to store fecal samples in the freezer immediately after collection until samples were delivered to the university and stored until analysis at −80°C. Fecal concentrations of acetate, propionate and butyrate were determined by gas chromatography-mass spectrometry (GC-MS). Frozen fecal samples (500 mg) was mixed 1:1 (weight: weight) with PBS (5 min) and afterward centrifuged at 14,000 x g for 10 min. Subsequently, 50 μL of supernatant was mixed with 650 μL of internal standard solution, containing methanol, internal standard (2 mg/mL 2-ethyl butyric acid) and formic acid (20%). The SCFA concentrations were determined through gas chromatography-mass spectrometry (GC-MS) (8890 GC System, Agilent Technologies) equipped with a PAL3 RSI 85 autosampler (Agilent). The temperature settings of the injector port, oven, flame ionization detector and mass spectrometer detector were 250°C, 200°C, 275°C and 225°C, respectively. Participants were also required to rate stool consistency of the sample using the Bristol stool scale.^79^ Data on self-reported gastrointestinal health are collected by a questionnaire based on the Rome III criteria.^80^ The questionnaire includes questions on presence of gastrointestinal complaints (i.e., abdominal pain, obstipation, bloating), defecation frequency, and stool consistency.

#### V4-16S rRNA amplicon and shotgun metagenomic sequencing

Gut microbiota composition was determined in both the fermentation pellets of the single donor *in vitro* fermentation experiment as well as fermentations with multiple donors for the Ussing chamber experiment. DNA was extracted by weighing 0.25 g of frozen feces into sterile 2.0 mL screw-cap tubes filled with 0.5 g of 0.1 mm zirconia beads and 5 glass beads of 2.5 mm diameter and adding 700 μL Stool Transport and Recovery (STAR) buffer (Roche Diagnostics, Almere, The Netherlands). Samples were subjected to repeated (3 × 1 min at 5.5 ms) bead-beating in a FastPrep-24™ 5G Instrument (MP Biomedicals, The Netherlands) followed by heating for 15 min at 95°C at 300 rpm and centrifuging for 5 min at 4°C at 16,100 x g. The supernatant was transferred into a sterile Eppendorf tube, the pellet was resuspended in 700 μL STAR and the cycle of bead-beating, heating and centrifuging was repeated. Supernatants were pooled and DNA was purified using a customized Maxwell® 16 Tissue LEV Total RNA purification Kit (XAS1220) on the Maxwell® 16 LEV Instrument (Promega, The Netherlands) and eluted in 50 μL nuclease free water (Qiagen, Hilden, Germany). DNA concentration was measured using a Qubit™ dsDNA Quantification BR Assay Kit on a Qubit Fluorometer (ThermoFisher Scientific, The Netherlands) and adjusted to 20 ng/μL with nuclease-free water. The V4 region of the 16S rRNA gene was amplified in duplicate using the barcoded primers 515F (5′-GTGYCAGCMGCCGCGGTAA-3′)^64^ and 806R (5′-GGACTACNVGGGTWTCTAAT-3′).^65^ Each 50 μL PCR reaction contained 10 μL 5x Phusion Green HF buffer (ThermoFisher Scientific, The Netherlands), 1 μL 10mM dNTPs (Promega, Madison, WI, United States), 0.5 μL Phusion Hot Start II DNA 2 U/μL polymerase (ThermoFisher Scientific, The Netherlands), 1 μL of each barcoded forward and reverse 10 μM primer, 1 μL of 20 ng/μL DNA template and 36.5 μL nuclease-free water. The PCR program consisted of an initial 30 s denaturation at 98°C for 10 min, followed by 25 cycles of 10 s denaturation at 98°C, 10 s annealing at 50°C, 10 s elongation at 72°C, and final extension for 420 s at 72°C. To verify the presence and size of each PCR product 2 μL were loaded onto a 2.2% agarose gel (Lonza Benelux B.V., Breda, The Netherlands) and run for 5 min at 200 V. The PCR products were pooled, further purified using the CleanPCR kit (CleanNA, Waddinxveen, The Netherlands) and the DNA concentration was again measured using Qubit. A library with an equimolar mix of purified PCR product, negative PCR and DNA extraction controls as well as positive mock communities (MC3 and MC4;^81^) was prepared and sent for Illumina Hiseq sequencing to Novogene (Novogene, The Netherlands). Raw amplicon sequences were processed using NG-Tax 2.0 with default settings but trimmed to 100 bp^82^ and resulting amplicon sequence variants (ASVs) were taxonomically annotated using the SILVA 138.1 database.^83^ For 16S rRNA gene amplicon sequencing, on average, we obtained 86,302 reads per sample, ranging from 10,746 to 200,239 reads. For shotgun metagenomic sequencing, DNA samples were diluted in 25 μL of nuclease-free water to a final concentration of >20 ng/μL. Metagenome sequencing (paired-end 150 bp) was perfromed using the Illumina NovaSeq 6000 platform by Novogene (Novogene, Cambridge, United Kingdom). Quality of raw metagenomic reads was checked using FastQC (version 0.12.1).^84^ All samples passed quality control with an average Phred score above 35 indicating high quality and suitability for downstream analysis. We obtained on average 50,232,230 metagenomic reads per sample, ranging from 30,610,398 to 203,879,552 reads. Read-based taxonomic profiling was done using MetaPhlAn 4 (version 4.0.6; default parameters) with the mpa_vJun23_CHOCOPhlAnSGB_202307 database^85^ and functional profiling using HUMAnN (version 3.0).^86^ Taxonomic profiles generated by MetaPhlAn 4 were used as input for HUMAnN and translated searches were performed against Uniref. 90 database.

#### Abdominal subcutaneous adipose tissue biopsy

Before and after the intervention (week 12) abdominal subcutaneous adipose tissue biopsy (up to ∼1 g) were collected 6–10 cm lateral from the umbilicus under local anesthesia (1% lidocaine) by needle biopsy after an overnight fast. The samples were washed with sterile saline to remove blood clots. A portion of tissue was fixed overnight at 4°C in 4% paraformaldehyde and embedded in paraffin for histological sections to determine adipocyte morphology. The remaining tissue was snap-frozen in liquid nitrogen and stored at −80°C for later analyses of targeted gene and protein expression. Histological sections (8 μm) were cut from paraffin-embedded tissue, mounted on microscope glass slides, and dried overnight in an incubator at 37°C. Sections were stained with hematoxylin and eosin. Digital images were captured with the use of a Leica DFC320 digital camera (Leica, Rijswijk, Netherlands) at ×20 magnification (Leica DM3000 microscope; Leica). Computerized morphometric analysis (Leica QWin V3, Cambridge, England) of individual adipocytes was performed by measuring at least 200 adipocytes per sample.

#### Body composition

Whole-body and regional fat mass, fat percentage, lean body mass, and bone mineral density were assessed using dual-energy X-ray absorptiometry scan using a 3-compartment model (Discovery A, Hologic, MUMC+, Maastricht, The Netherlands). In addition, fasting body weight (in underwear using a calibrated weight scale) and height (barefoot) were measured to calculate BMI.

#### Intrahepatic lipid content

IHL content was quantified by ^1^H-MRS after an overnight fast. All 1H-MRS experiments were performed on a 3-T MR system (Achieva 3 T-X Philips Healthcare) by using a 32-channel sense cardiac/torso coil (Philips Healthcare). A volume of interest of 30 × 30 × 30 mm was placed in the lower hepatic lobe, and a Stimulated Echo Acquisition Mode (STEAM) (repetition time [TR] = 4500 ms, echo time [TE] = 20 ms, mixing time (TM) = 16 ms, spectral bandwidth 2 kHz, number of acquired data points 2048, number of signal averages = 128) sequence was used, as described previously.^87^ Participants were asked to breathe in the 4-s rhythm of the spectroscopic measurement to prevent motion artifacts. Variable power and optimized relaxation delays (VAPOR) water suppression was applied, and an additional water reference scan was obtained (number of signal averages = 16). All spectra were post-processed with a custom-written MATLAB script (MATLAB 2014b, The MathWorks, Inc.). Single acquisitions were separately phased and frequency-aligned before averaging, and spectra were corrected for eddy currents. Lipid peaks were fitted with a custom-developed algorithm based on prior knowledge that was defined earlier in *in vitro* studies.^87^ Values were corrected for T2 relaxation, and the CH_2_ resonance, as a percentage of the sum of CH_2_ + H_2_O resonances (CH_2_/(CH_2_ + water)), was used as a parameter of IHL content.

### Quantification and statistical analysis

The calculated sample size was based on a 20% physiologically relevant change of insulin sensitivity (2.86μmol/kg/min) in our primary outcome parameter peripheral insulin sensitivity measured via the Hyperinsulinemic-euglycemic clamp technique. A power of 80%, an α value of 0.05, a within-group residual SD of 2.70^54^ and a 22% dropout rate was assumed, resulting in a sample size of *n* = 21 per intervention group. A total of 35 participants (*n* = 17 intrinsic fiber, *n* = 18 placebo) were included in the final analysis unless otherwise stated in legends of figures and tables. All data were evaluated for normality. Model assumptions were tested by plotting residual and predicted values and by visually inspecting residual Q-Q plots, to test homogeneity of variances and normality of residuals, respectively. Two-tailed *p* < 0.05 was considered statistically significant. Linear Mixed Model Analysis was used to analyze difference between treatments with intervention group (intrinsic fiber versus placebo) and time (pre versus post or week 0 versus 6 and 12) and, and their interaction (Group x Time) as fixed effects and individual as random effect using IBM SPSS v28. Post-hoc analysis for differences between time points was done using Fisher’s least signficance difference test following significant results for the Group × Time interaction. Prior to conducting the analysis, heteroscedastic data points were log transformed to reduce skewness and approximate a normal distribution. Following these adjustments, normalization techniques were implemented to further align the data with a Gaussian distribution to meet the assumptions of normality required for statistical analyses. Adipocyte frequency distribution was categorized into 4 distinct sizes: <50μm, 50-69μm, 70-80μm and >90μm. Chi-squared analysis indicated a significant difference between the groups for these size categories *p* < 0.001. Post-hoc analysis conducted with pairwise comparisons using multiple z-tests of two proportions and Bonferroni correction with *p* < 0.0125 considered significant. Bowel function outcomes and gut microbiota data were analyzed using R version 4.2.3. Changes in stool consistency were assessed using linear mixed modeling as implemented in the lme4^88^ and lmerTest^89^^,^^90^ package with the intervention group, time (pre versus or post or week 0 versus 2, 6, 9 or 12), and their interaction as fixed effects and individual as a random effect. Estimated marginal means were calculated using the emmeans package.^91^ Bowel function outcomes (stool frequency and gastrointestinal symptoms) at baseline, 2 weeks, 6 weeks, 9 weeks, and 12 weeks were analyzed using the Friedman test due to assumption violation for linear repeated measures testing, and if applicable, pairwise comparisons were calculated using paired Wilcoxon test with false-discovery rate (FDR) correction as implemented in the rstatix package. Missing values in bowel function outcomes were imputed using the median of the respective intervention group’s time period. Gut microbiota outcomes at baseline, 2 weeks, 6 weeks, 9 weeks, and 12 weeks based on 16S rRNA gene amplicon sequencing were analyzed as described previously^18^ using the R packages mare^92^ as well as the vegan, phyloseq,^93^ microbiome^94^ and microViz.^95^ Multivariate community analysis was done using Principal Coordinate Analysis (PCoA) based on Bray-Curtis dissimilarity (β-diversity). For univariate analysis at the genus level, taxa counts were converted into relative abundances (%), and differential abundance testing with FDR correction was performed as implemented in mare. Shotgun metagenomic profiles from baseline and 12 weeks were used to assess species-level information of taxa of interest. MetaPhlan obtained relative abundances were analyzed using similar methods as for 16S rRNA gene amplicon sequences.^96^ Further responder analysis was performed by dividing he intrinsic fiber group based on the observed improvement in the primary study outcomes (insulin-mediated glucose disposal) of more than 15% (high responders) or less than 15% (low responders). Baseline and endpoint differences in gut microbiota composition were then assessed using the same data analytical approaches as described above. The used statistical tests and P-values are indicated in (the legends of) Figures and Tables as well as in the results section.

#### Additional resources

This trial was registered at **ClinicalTrials.gov:** **NCT04714944**.

## Supplemental information

Document S1. Figures S1–S8 and Tables S1–S8 and S11–S15

Table S9. Changes in mean relative abundances of common taxa (mean relative abundance of at least 1% and mean prevalence in at least 50% of the samples) in the treatment group from baseline (week 0) over time and their respective fold changes, *p* values and FDR-corrected *q* values

Table S10. Changes in mean relative abundances of common taxa (mean relative abundance of at least 1% and mean prevalence in at least 50% of the samples) in the placebo group from baseline (week 0) over time and their respective fold changes, *p* values and FDR-corrected *q* values

Document S2. Article plus supplementary information
