# Supplementary material for: Intrinsic chicory root fibers modulate colonic microbial butyrate-producing pathways and improve insulin sensitivity in individuals with obesity
Source: Cell Rep Med. 2025 Jul 15;6(7):102237. doi: 10.1016/j.xcrm.2025.102237 (PMC12281436; doi:10.1016/j.xcrm.2025.102237)
Supplement: Document S1. Figures S1–S8 and Tables S1–S8 and S11–S15 [file mmc1.pdf]

Cell Reports Medicine, Volume *6*

Supplemental information

Intrinsic chicory root fibers modulate colonic microbial butyrate-producing pathways and improve insulin sensitivity in individuals with obesity

Lina Omary, Emanuel E. Canfora, Marie-Luise Puhlmann, Asimenia Gavriilidou, Iris Rijnaarts, Jens J. Holst, Yvonne M.H. Op den Kamp-Bruls, Willem M. de Vos, and Ellen E. Blaak

Supplementary Material

Supplementary figures

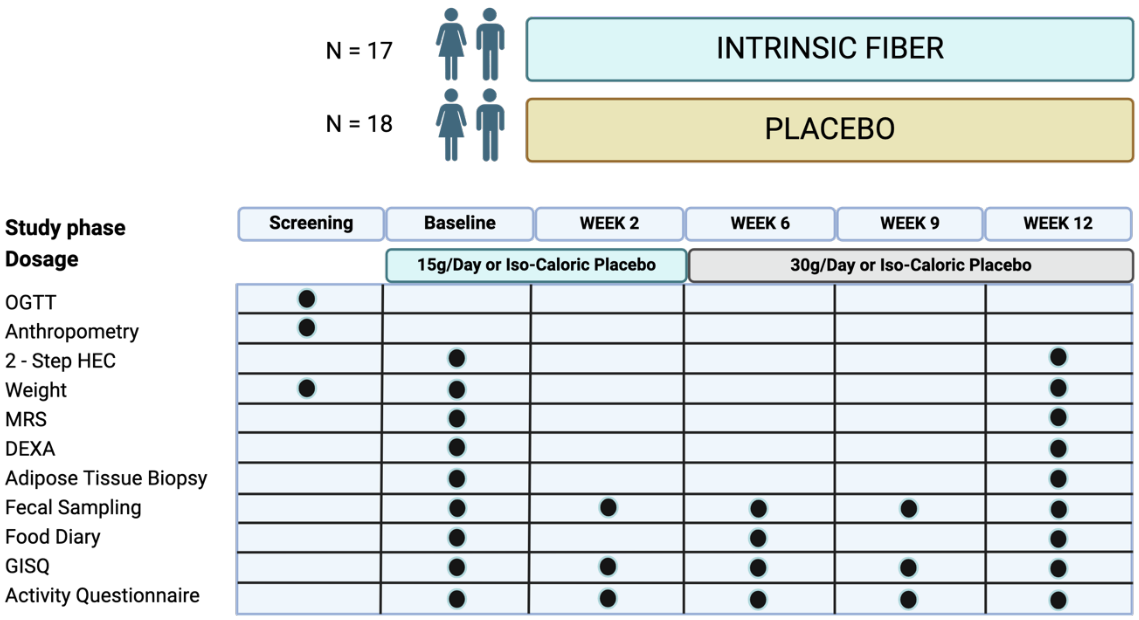

Figure S1 Study design of the randomized, placebo-controlled parallel trial investigating the effects of an intrinsic fiber product on peripheral insulin sensitivity and the gut microbiota. Circles denote measurement time points for every outcome. OGTT; Oral glucose tolerance test, DEXA; Dual X-Ray Absorptiometry, MRS; Magnetic Spectroscopy, GISQ; Gastrointestinal Symptoms Questionnaire.

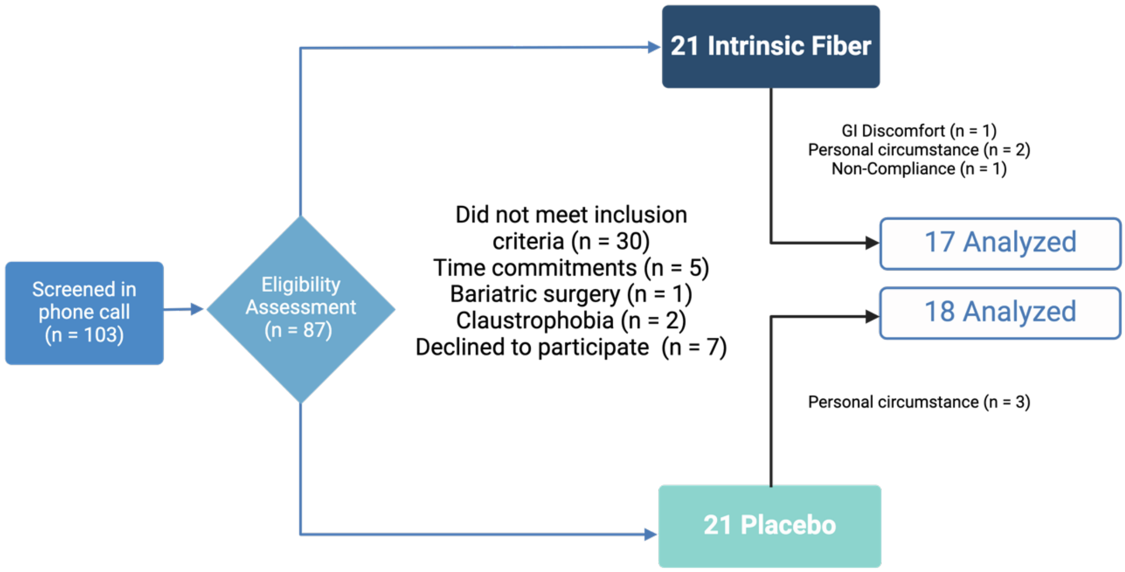

Figure S2. CONSORT flow diagram. Participant flow during the process of recruitments, randomization, and data analysis in the study.

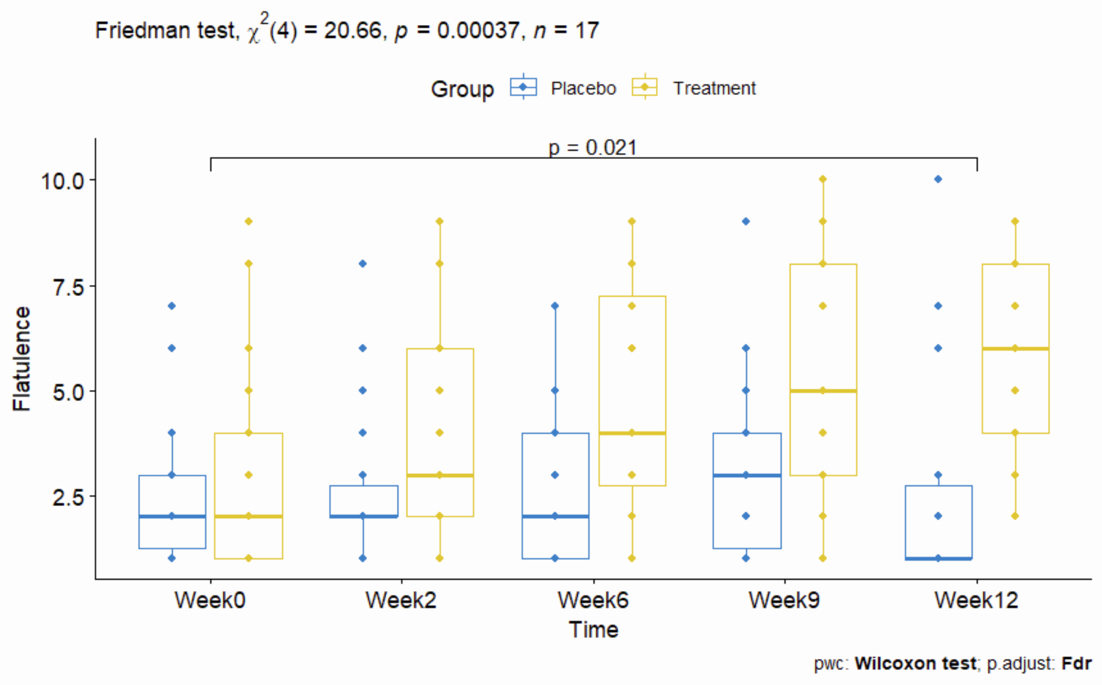

Figure S3. Statistically significant changes in flatulence between baseline (week 0) and end of the study (week 12) following dried chicory root intake (treatment group) versus placebo.

Data are indicated as median and interquartile range

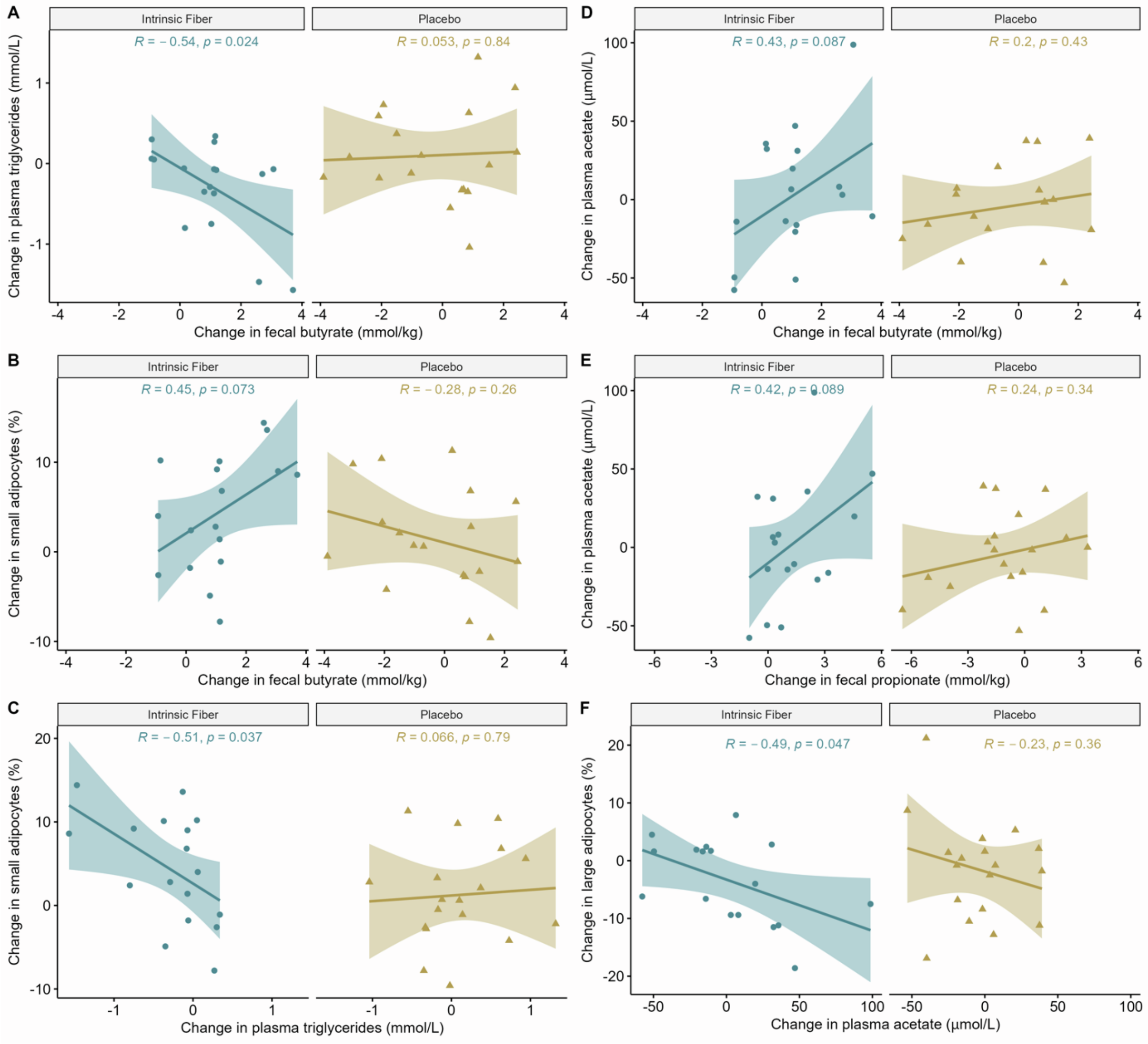

Figure S4. Spearman correlations between changes at 12 weeks in metabolic health markers, fecal and plasma short-chain fatty acids, showing moderate relations between outcome variables. (A) Correlation between increases in fecal butyrate (mmol/kg) and decreases in plasma triglycerides (mmol/L) levels. (B) Correlation between increases in fecal butyrate levels (mmol/kg) and change in % small adipocytes . (C) Correlation between decreases in plasma triglyceride levels (mmol/L) and increases in % small adipocytes (D) Correlation between increases in fecal butyrate (mmol/kg) and increases in plasma acetate (µmol/L) levels. (E) Correlation between increases in fecal propionate (mmol/kg) and increases in plasma acetate (µmol/L) levels. (F) Correlation between change in plasma acetate (µmol/L) and change in % of large adipocytes.

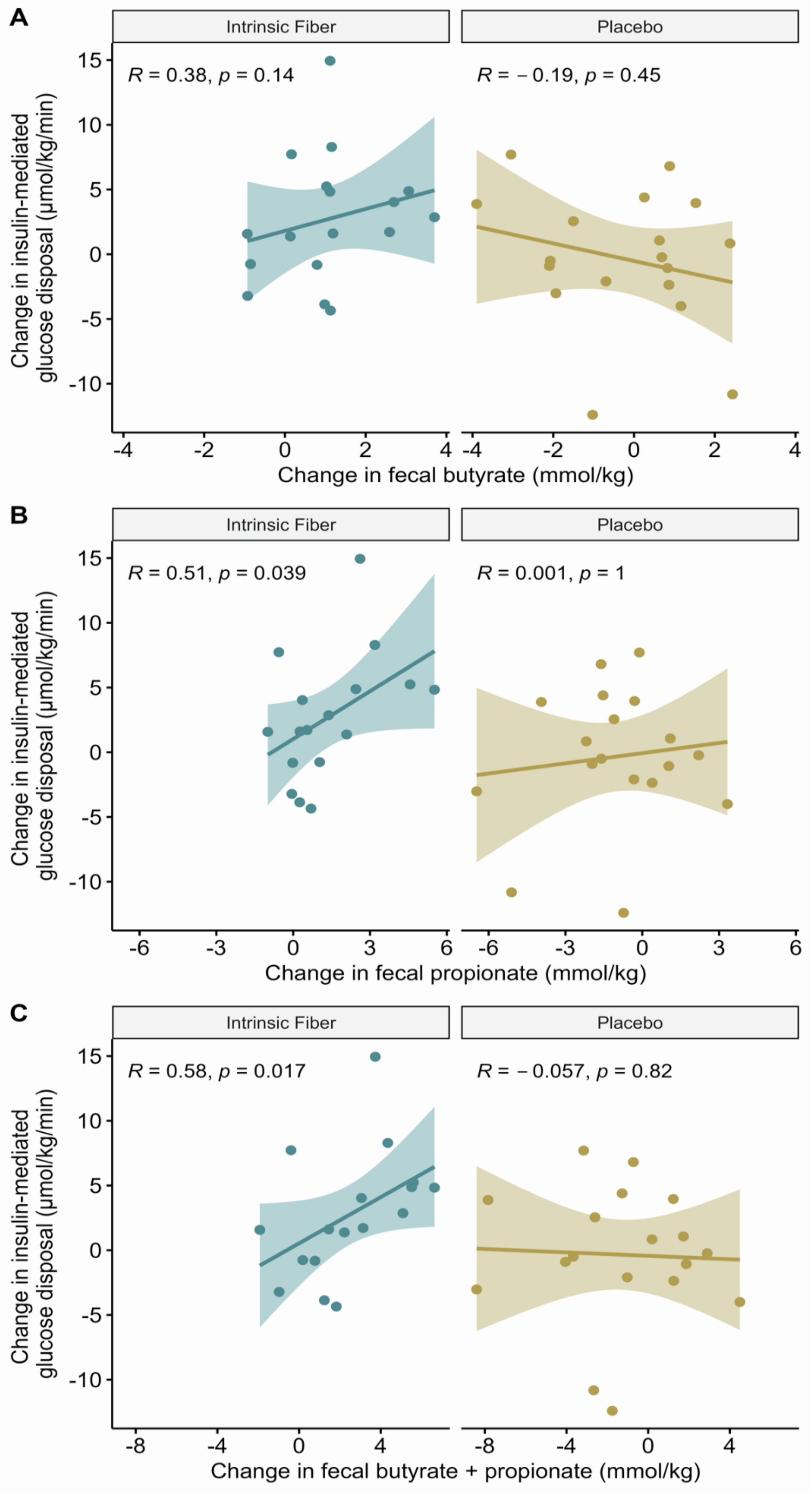

Figure S5: Spearman correlations between change in fecal butyrate (A) and propionate (B) as well the sum of fecal butyrate and propionate (C) and the change in insulin-mediated glucose disposal in the intrinsic fiber group and in the control group

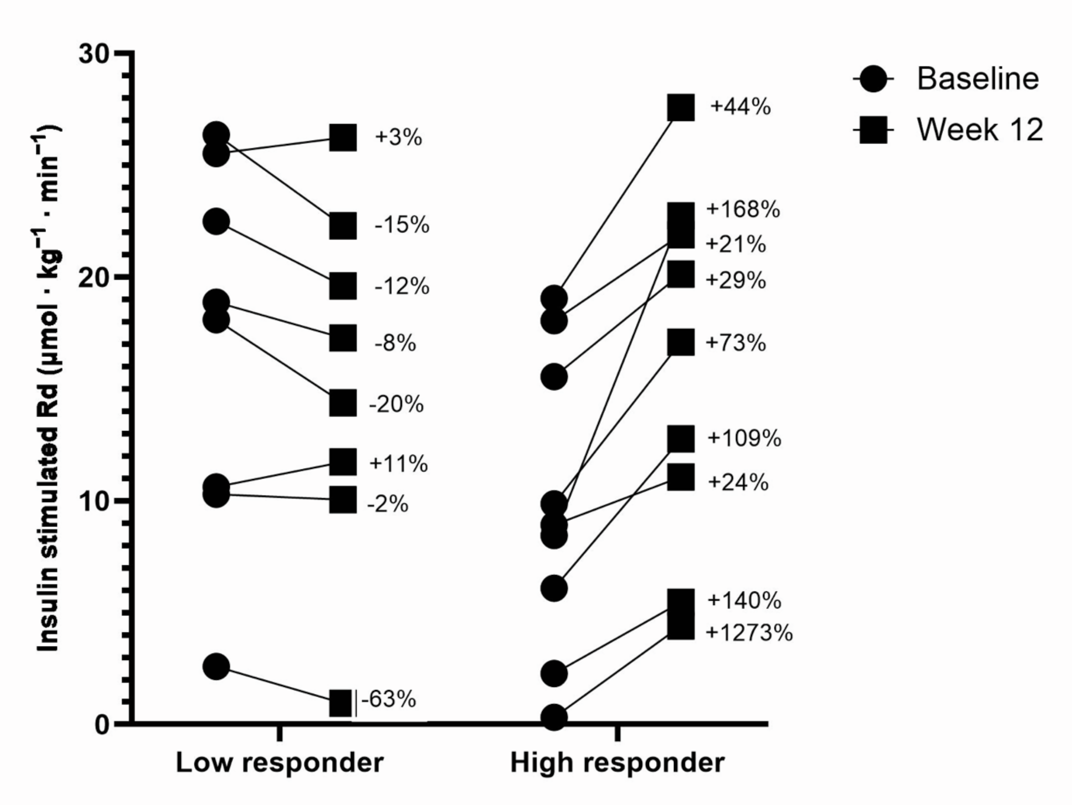

Figure S6: Division in Low responders and High responders in the intrinsic fiber group based on change in insulin-mediated glucose disposal.Absolute changes are plotted with the percentage change indicated.

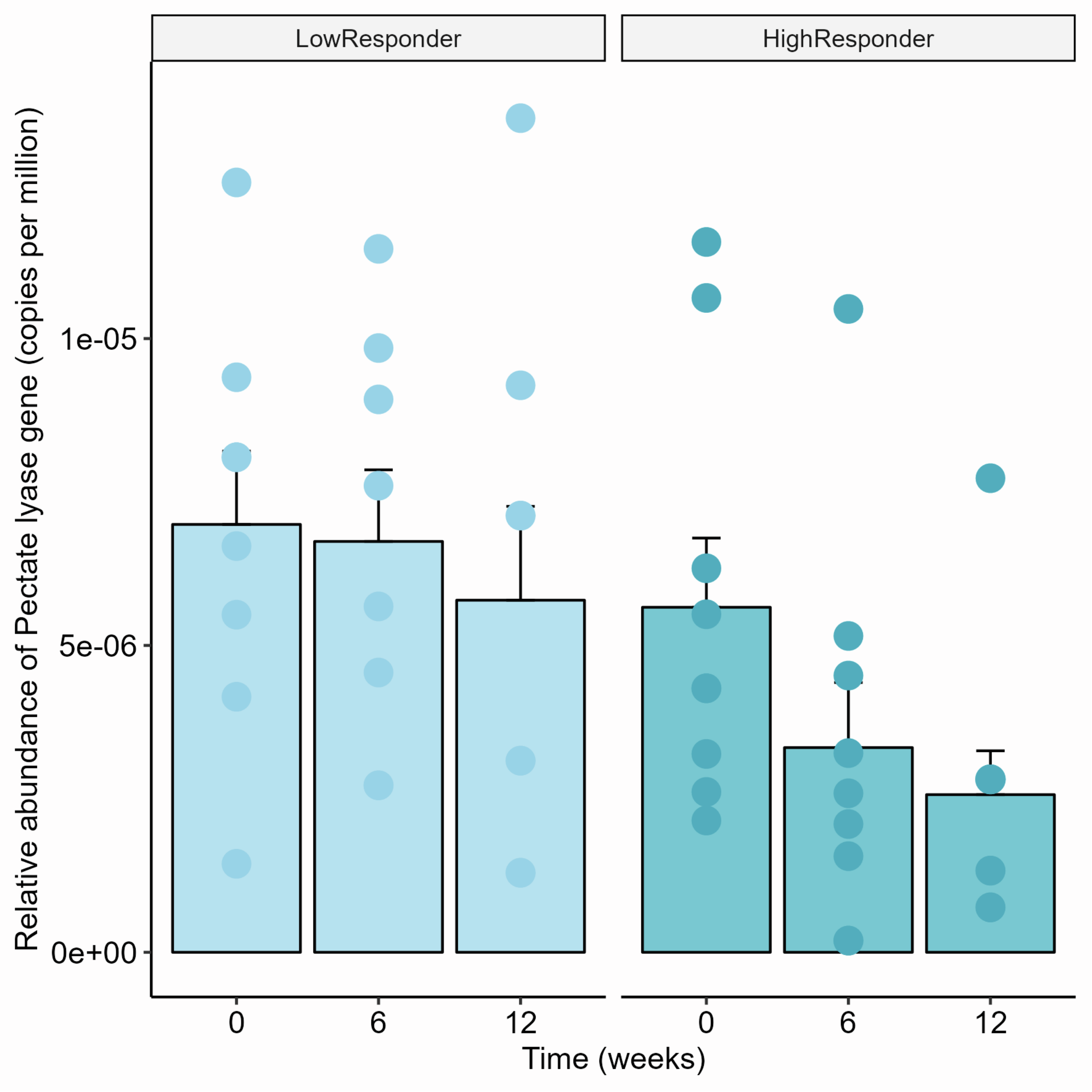

Figure S7. Relative abundance of Pectate lyase gene in low and high responders at baseline (week 0), week 6 and week 12.

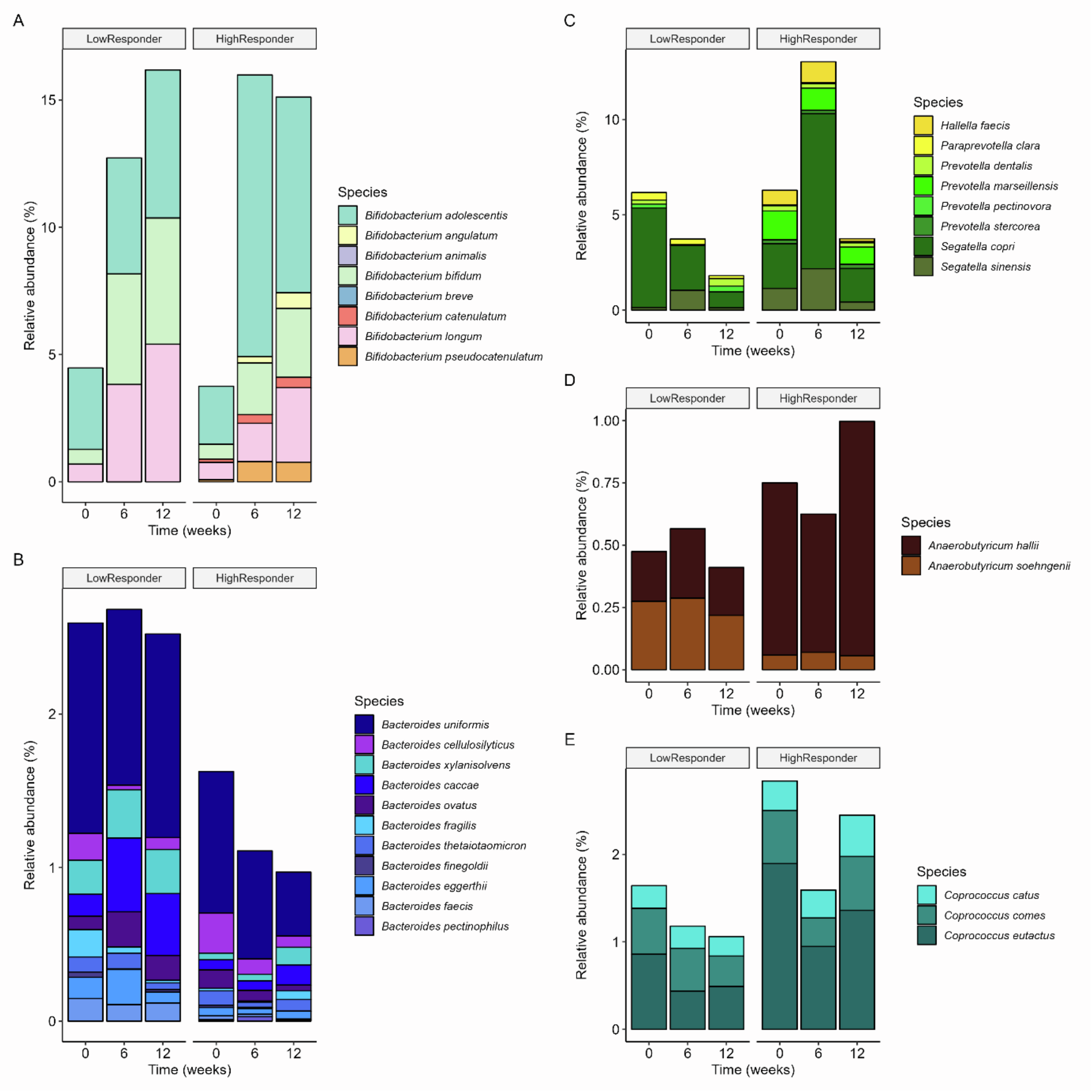

Figure S8. Detected species based on taxonomic profiling using metagenomics in low and high responders at baseline (week 0), week 6 and week 12. (**A**) *Bifidobacterium* spp., (**B**) *Bacteroides* spp., (**C**) *Prevotella* spp., (**D**) *Anaerobutyricum* spp. (formerly *Eubacterium hallii group*), (**E**) *Coprococcus* spp.

Supplementary Tables

Table S1: Nutritonal composition of the Organic puffed millet (placebo) and WholeFiber^{TM} product (intrinsic fiber group)

**Nutritional value (per 100g)**	**Organic puffed millet**	**WholeFiber^{TM}**
Energy (Kcal)	381	196
Protein (g)	11.0	4
Fat (g)	4.3	0
Carbohydrates (g)	72.8	5
Dietary fiber (g)	3.5	85

Table S2. Compliance

Compliance Measure	Intrinsic Fiber (n=17)	Placebo (n=18)	P-value
Total Empty Bags Returned /168	134.3 ± 24.5	141.3 ± 26.3	0.386
Total bags consumed according to intake log	164.1 ± 17.7	164.7 ± 11.3	0.809
No. of days under treatment	89 ± 5.8	88.8 ± 3.5	0.186
Compliance (%)	96.4	98.1	0.383

Accountability of product intake was assessed in duplicate. At the end of the study participants were required to return all sachets which contained the product. The sachet count was then cross-referenced to the intake logged in the participants personal intake log to ensure both groups consumed the product two times a day for a maximum of 90 days.

Table S3. Adverse events

Adverse Event		Intrinsic Fiber (n = 17)	Placebo (n = 18)
Infections	Flu (undefined)	3	
	UTI		1
COVID-related events	Vaccine Side-effects	2	3
	Upper respiratory tract infection	1	1
Other	Physical Injury	1	

Table S4. Stool softness outcomes measured by using the Bristol Stool Form Scale (BSFS) over time in the treatment (dried chicory root) and placebo group.

	Time	Intrinsic Fiber (n=17)	Placebo (n=18)	P-value
Stool Softness (BSFS)	WEEK 0	3.97 (0.37)	4.08 (0.36)	
	WEEK 2	3.94 (0.37)	4.17 (0.36)	0.847
	WEEK 6	4.50 (0.37)	4.11 (0.36)	0.392
	WEEK 9	4.16 (0.38)	3.86 (0.36)	0.488
	WEEK 12	4.35 (0.37)	4.22 (0.36)	0.678

Data are represented as mean± SEM. The p-value represents the difference in change between intervention groups per week as assessed using linear mixed modeling.

Table S5. Stool frequency and gastrointestinal outcome scores over time in the treatment (dried chicory root) and placebo group.

	Intrinsic Fiber (n=17)						Placebo (n=18)					
	**Week 0**	**Week 2**	**Week 6**	**Week 9**	**Week 12**	**Friedmann Test**	**Week 0**	**Week 2**	**Week 6**	**Week 9**	**Week 12**	**Friedmann Test**
Stool Frequency (x/day)	1.00 [1.00, 2.00]	2.00 [1.00, 2.00]	2.00 [1.00, 2.50]	2.00 [1.00, 2.50]	2.00 [1.00, 3.00]	$\chi^2(4) = 5.09$, $p = 0.278$	1.00 [1.00, 1.00]	1.00 [1.00, 2.00]	1.00 [1.00, 1.00]	1.00 [1.00, 1.75]	1.00 [1.00, 2.00]	$\chi^2(4) = 2.97$, $p = 0.563$
Flatulence score	2.00 [1.00, 4.00]	3.00 [2.00, 6.00]	4.00 [2.75, 7.25]	5.00 [3.00, 8.00]	6.00 [4.00, 8.00]	$\chi^2(4) = 20.66$, $p < 0.001$	2.00 [1.25, 3.00]	2.00 [2.00, 2.75]	2.00 [1.00, 4.00]	3.00 [1.25, 4.00]	1.00 [1.00, 2.75]	$\chi^2(4) = 4.48$, $p = 0.344$
Bloating score	1.00 [1.00, 2.00]	1.00 [1.00, 3.00]	1.00 [1.00, 2.50]	1.00 [1.00, 2.00]	2.00 [1.00, 3.00]	$\chi^2(4) = 5.47$, $p = 0.242$	1.00 [1.00, 2.00]	1.00 [1.00, 2.00]	2.00 [1.00, 3.00]	1.00 [1.00, 2.75]	1.00 [1.00, 2.75]	$\chi^2(4) = 5.60$, $p = 0.231$
Rumbling score	1.00 [1.00, 2.00]	2.00 [1.00, 3.00]	2.00 [1.00, 4.00]	2.00 [1.00, 3.50]	2.00 [1.00, 2.00]	$\chi^2(4) = 3.46$, $p = 0.485$	1.00 [1.00, 2.00]	1.00 [1.00, 2.00]	1.00 [1.00, 3.00]	1.00 [1.00, 2.00]	1.00 [1.00, 1.00]	$\chi^2(4) = 8.61$, $p = 0.072$
Cramping score	1.00 [1.00, 2.00]	1.00 [1.00, 1.00]	1.00 [1.00, 1.25]	1.00 [1.00, 1.00]	1.00 [1.00, 1.00]	$\chi^2(4) = 0.92$, $p = 0.922$	1.00 [1.00, 1.00]	1.00 [1.00, 1.00]	1.00 [1.00, 1.00]	1.00 [1.00, 1.75]	1.00 [1.00, 1.00]	$\chi^2(4) = 2.38$, $p = 0.667$
Regurgitation score	1.00 [1.00, 1.00]	1.00 [1.00, 1.00]	1.00 [1.00, 2.00]	1.00 [1.00, 1.00]	1.00 [1.00, 1.00]	$\chi^2(4) = 6.33$, $p = 0.176$	1.00 [1.00, 1.38]	1.00 [1.00, 1.75]	1.00 [1.00, 2.75]	1.00 [1.00, 2.00]	1.00 [1.00, 1.75]	$\chi^2(4) = 6.17$, $p = 0.187$

Data are represented as median and range. Statistics was done by a Friedman test (Chi-square test χ^2)

Table S6. Difference in physical activity levels between intrinsic fiber and placebo intervention after 12 weeks.

**Activity Measure**	**Intrinsic Fiber (n=17)**			**Placebo (n=18)**			**P-value**
	Week 0	Week 6	Week 12	Week 0	Week 6	Week 12	Group x Time
Total (active days/week)	3.14 ± 0.61	4.00 ± 0.56	4.06 ± 0.57	4.56 ± 0.60	4.28 ± 0.56	3.94 ± 0.57	0.165
Light Intensity (minutes/week)	1137.07 ± 210	1151.67 ± 205	1121.94 ± 193	721.39 ± 209	819.72 ± 205	649.44 ± 193	0.785
Medium Intensity (minutes/week)	791.05 ± 170	996.94 ± 131	914.44 ± 151	724.44 ± 168	557.50 ± 131	652.22 ± 152	0.182
High Intensity (minutes/week)	22.96 ± 51.56	20.00 ± 54.36	33.50 ± 57.27	175.28 ± 51.2	143.33 ± 54	159.44 ± 57.2	0.771
Total Activity (minutes/week)	1921.11 ± 285	2168.61 ± 252	2069.89 ± 249	1621.11 ± 285	1520.56 ± 252	1461.11 ± 249	0.327

Data are Mean ± SEM. Minutes per week spent in different categories of physical activity. There were no significant differences between the groups before, during or after intervention assessed using a validated questionnaire for physical activity assessment. Data analyzed using linear-mixed model analysis representing Group x Time interactions.

Table S7. Dietary intake as assessed by self-reported 3-day food record pre, mid-way and post intervention

**Macronutrient**	**Intrinsic Fiber (n=17)**			**Placebo (n=18)**			**P-value**
	Week 0	Week 6	Week 12	Week 0	Week 6	Week 12	Group x Time
Avg. Daily Intake (MJ)	8.94 ± 0.64	8.92 ± 0.84	8.26 ± 0.66	7.84 ± 0.63	7.82 ± 0.82	7.92 ± 0.65	0.392
Avg. Daily Intake (Kcal)	2188.5 ± 152	2087.8 ± 193	1968.3 ± 169	1932.2 ± 147	1947.3 ± 190	1898.5 ± 164	0.562
Water (g/d)	2081.6 ± 168	1837.8 ± 173	1800.2 ± 119	1912.5 ± 163	1984.7 ± 170	1938.4 ± 116	0.243
Protein (g/d)	87.7 ± 6.7	84.3 ± 8.2	88.5 ± 7.5	77.8 ± 6.6	82.0 ± 8.1	80.9 ± 7.3	0.674
Fat (g/d)	92.8 ± 7.9	92.8 ± 10	81.2 ± 9.1	73.5 ± 7.7	74.2 ± 9.8	76.7 ± 8.8	0.128
Saturated Fat (g/d)	34.29 ± 3.5	33.59 ± 4.6	30.30 ± 4	26.27 ± 3.4	28.33 ± 4.5	29.60 ± 3.9	0.156
Carbohydrates (g/d)	216.3 ± 19	208.1 ± 21.5	190.4 ± 16.5	181.5 ± 18.1	195.8 ± 21.1	182.7 ± 16	0.374
Fiber (g/MJ)	2.52 ± 0.18	2.26 ± 0.16	2.17 ± 0.18	2.80 ± 0.18	2.57 ± 0.16	2.58 ± 0.18	0.846

Data are Mean ± SEM. Data analyzed using linear-mixed model analysis representing Group x Time interactions. Linear mixed model analysis showed that there were no significant differences between the groups before, during or after intervention for any macronutrient.

Table S8. The effects of 12-weeks of Intrinsic Fiber or Placebo intervention on body composition as measured by DXA

	Intrinsic Fiber (n = 17)		Placebo (n = 18)		P-value
	**Week 0**	**Week 12**	**Week 0**	**Week 12**	Group x Time
Weight (kg)	97.4 ± 3.14	96.4 ± 3.21	95.4 ± 3.70	94.9 ± 3.89	0.559
BMI	32.3 ± 1.01	32.0 ± 1.04	31.7 ± 0.62	31.5 ± 0.70	0.493
Fat total (%)	34.09 ± 1.95	33.75 ± 1.95	33.99 ± 1.70	34.23 ± 1.70	0.282
Fat mass total (kg)	33.35 ± 2.12	32.99 ± 1.96	32.63 ± 1.91	32.96 ± 2.18	0.332
Lean mass total (kg)	62.82 ± 2.89	62.87 ± 3.24	61.41 ± 2.99	61.19 ± 3.30	0.732
VAT mass (g)	1016.77 ± 66.03	1025.72 ± 79.40	893.41 ± 55.01	915.65 ± 72.15	0.806

Data are Mean ± SEM. There were no significant differences between the groups after intervention (Post) compared to baseline (Pre) for any variable of body composition. DXA; Dual X-Ray Absorptiometry, VAT; Visceral adipose tissue. Data analyzed using linear-mixed model analysis representing Group x Time interactions.

Table S11. Insulin Senstivity Indices before (baseline) Insulin Infusion, and during the Low Insulin Infusion and High Insulin Infusion Rate of the 2-Step Hyperinsulinemic-Euglycemic Clamp Method.

Measurement	Step	Intrinsic Fiber (n=17)		Placebo (n=18)		P-value
		**Week 0**	**Week 12**	**Week 0**	**Week 12**	Group x Time
**GIR (mg · kg^{-1} · min^{-1})**	Low insulin	0.82 ± 0.15	0.95 ± 0.15	1.04 ± 0.22	0.90 ± 0.17	0.156
	High insulin	4.60 ± 0.40	5.14 ± 0.41	4.58 ± 0.47	4.52 ± 0.58	0.068
**M-value (mg · kg^{-1} · min^{-1})**	Low insulin	0.84 ± 0.16	0.88 ± 0.11	1.17 ± 0.26	0.88 ± 0.21	0.160
	High insulin	4.55 ± 0.43	5.17 ± 0.37	4.62 ± 0.46	4.41 ± 0.51	0.032
**Ra (*μ*mol · kg^{-1} · min^{-1})**	Baseline	11.54 ± 0.29	11.66 ± 0.30	11.90 ± 0.35	11.93 ± 0.30	0.792
	Low insulin	9.76 ± 0.53	10.28 ± 0.51	10.75 ± 0.88	10.03 ± 0.56	0.081
	High insulin	24.42 ± 1.92	26.92 ± 1.93	25.00 ± 2.47	23.93 ± 2.92	0.079
	Δhigh ins - baseline	12.88 ± 1.96	15.56 ± 1.91	13.09 ± 2.47	12.00 ± 2.94	0.088
**Rd (*μ*mol · kg^{-1} · min^{-1})**	Baseline	11.76 ± 0.29	11.88 ± 0.30	12.12 ± 0.35	12.15 ± 0.30	0.794
	Low insulin	10.02 ± 0.53	10.55 ± 0.51	11.02 ± 0.89	10.30 ± 0.57	0.080
	High insulin	24.87 ± 1.94	27.40 ± 1.95	25.45 ± 2.49	24.37 ± 2.95	0.078
	Δhigh ins - baseline	13.11 ± 1.98	15.52 ± 1.92	13.33 ± 2.50	12.22 ± 2.98	0.085
**NOGD (*μ*mol · kg^{-1} · min^{-1})**	Baseline	10.15 ± 0.39	9.67 ± 0.37	9.53 ± 0.62	10.56 ± 0.61	0.068
	High insulin	15.74 ± 1.52	18.21 ± 1.66	16.26 ± 2.05	16.09 ± 2.44	0.108
	Δhigh ins - baseline	5.60 ± 1.45	8.54 ± 1.82	6.73 ± 2.12	5.54 ± 2.43	0.027
**EGP (*μ*mol · kg^{-1} · min^{-1})**	Baseline	11.54 ± 0.29	11.66 ± 0.30	11.90 ± 0.35	11.93 ± 0.30	0.792
	Low insulin	5.22 ± 0.46	5.02 ± 0.52	4.98 ± 0.54	5.04 ± 0.42	0.669
	High insulin	0.24 ± 0.14	0.43 ± 0.26	0.56 ± 0.26	0.39 ± 0.20	0.756
	% suppression low vs baseline	55.37 ± 3.33	57.51 ± 3.78	59.07 ± 3.97	58.06 ± 3.00	0.489

Values are shown as Mean ± SEM. Data analyzed using linear-mixed model analysis representing Group x Time interactions. P-values <0.05 are considered significant. *EGP;* endogenous glucose production, *GIR;* glucose infusion rate*, NOGD;* non-oxidative glucose disposal, *Ra;* rate of glucose appearance, *Rd;* rate of glucose disappearance

Table S12. Substrate Oxidation, Respiratory Quotient and Energy Expenditure at resting, low insulin infusion and high insulin infusion rate of 2-Step Hyperinsulinemic-Euglycemic Clamp Method.

Measurement	Step	Intrinsic Fiber (n=17)		Placebo (n=18)		P-value
		pre	post	pre	post	Group x Time
RER	Baseline	0.746 ± 0.009	0.754 ± 0.008	0.762 ± 0.009	0.747 ± 0.008	0.058
	Low insulin	0.789 ± 0.007	0.793 ± 0.009	0.800 ± 0.007	0.787 ± 0.009	0.177
	High insulin	0.86 ± 0.009	0.86 ± 0.011	0.87 ± 0.009	0.85 ± 0.01	0.163
Energy Expenditure	Baseline	5.16 ± 0.18	5.17 ± 0.18	4.89 ± 0.18	4.93 ± 0.17	0.780
	Low insulin	5.05 ± 0.19	4.93 ± 0.16	4.88 ± 0.19	4.81 ± 0.16	0.661
	High insulin	5.24 ± 0.17	5.14 ± 0.16	4.89 ± 0.17	4.97 ± 0.16	0.075
CHO Oxidation (g/min)	Baseline	0.029 ± 0.01	0.038 ± 0.009	0.043 ± 0.01	0.028 ± 0.009	0.082
	Low insulin	0.076 ± 0.008	0.079 ± 0.01	0.082 ± 0.008	0.069 ± 0.009	0.265
	High insulin	0.156 ± 0.010	0.159 ± 0.012	0.154 ± 0.010	0.139± 0.012	0.304
FAT Oxidation (g/min)	Baseline	0.097 ± 0.005	0.093 ± 0.004	0.085 ± 0.005	0.092 ± 0.004	0.103
	Low insulin	0.076 ± 0.005	0.072 ± 0.005	0.069 ± 0.005	0.073 ± 0.005	0.221
	High insulin	0.048 ± 0.005	0.045 ± 0.005	0.042 ± 0.005	0.049 ± 0.005	0.099

Values are shown as Mean ± SEM. Data analyzed using linear-mixed model analysis representing Group x Time interactions. P-values <0.05 are considered significant. CHO; Carbohydrate, EE, Energy Expenditure, RER; Respiratory Exchange Ratio, Substrate oxidation, Respiratory Exchange Ratio and Energy Expenditure was analyzed using OMNICAL Ventilate Hood System during the last 30 minutes of each steady state measurement of the 2-step clamp method.

Table S13. The effects of 12-weeks of Intrinsic Fiber or Placebo intervention on fasting circulating plasma metabolites and hormones.

**Week 0**	**Week 12**			**Week 0**			**Week 12**
	pre	post	Within Group P-value	pre	post	Within Group P-value	Group x Time
Glucose mmol/L	5.94 ± 0.10	5.87 ± 0.11	0.283	5.88 ± 0.17	6.00 ± 0.16	0.060	0.040
Insulin mU/L	5.00 ± 0.84	5.18 ± 0.82		8.95 ± 2.11	10.1 ± 2.65		0.460
PYY (pmol/l)	8.06 ± 1.40	8.35 ± 1.19		9.06 ± 1.23	8.61 ± 1.77		0.683
GLP-1 (pmol/l)	8.06 ± 0.64	7.53 ± 0.54		8.33 ± 0.68	6.83 ± 0.70		0.352
HbA1c (%)	5.72 ± 0.08	5.69 ± 0.08		5.67 ± 0.07	5.73 ± 0.07		0.102
Total cholesterol (mmol/l)	4.99 ± 0.22	4.96 ± 0.16		5.04 ± 0.17	4.92 ± 0.17		0.453
HDL (mmol/l)	1.04 ± 0.07	1.05 ± 0.07		1.15 ± 0.05	1.18 ± 0.05		0.190
TG (mmol/l)	2.00 ± 0.24	1.71 ± 0.15	0.041	1.74 ± 0.16	1.84 ± 0.22	0.457	0.049
TNF-α (pg/ml)	1.32 ± 0.08	1.36 ± 0.09		1.28 ± 0.07	1.33 ± 0.08		0.870
IL-8 (pg/ml)	8.27 ± 0.42	8.18 ± 0.60		6.95 ± 0.42	7.14 ± 0.49		0.686
IL-6 (pg/ml)	1.38 ± 0.28	1.18 ± 0.20		0.92 ± 0.07	0.99 ± 0.09		0.141
IL-10 (pg/ml)	0.24 ± 0.02	0.23 ± 0.02		0.23 ± 0.02	0.24 ± 0.04		0.546
IFN-g (pg/ml)	8.52 ± 2.39	6.49 ± 0.93		5.81 ± 0.75	6.97 ± 1.17		0.246

Values are shown as Mean ± SEM. GLP-1; Glucagon-Like Peptide -1, HDL; High-Density Lipoprotein, IL-6, 8, 10; Interleukin 6, 8, 10, IFN-γ; Interferon-gamma, PYY; Peptide YY, TG; Triglycerides, TNF-α; Tumor-Necrosis Factor alpha. P-values for overall intervention effect (Group x Time interaction) assessed by linear mixed modelling after 12 weeks of intervention between groups. P-values for between time-point differences within groups assessed by Fisher's least significant testing for significant Group x Time interaction p-values. P-values <0.05 are considered significant.

Table S14 Baseline differences between low compared to high responders based on peripheral insulin sensitivity.

	High Responders	Low Responders	p-value
Sex, male/female	3/6	4/4	0.839
Age, y	59.1 ±5.1	64.9 ±5.5	0.040
Weight, kg	99.5 ±11.8	95.0 ±14.6	0.495
Height, m	1.75 ±0.07	1.71±0.10	0.326
Body mass index, kg/m^2	32.2 ±3.7	32.5 ±4.9	0.906
Waist-Hip-Ratio	1.00 ±0.10	0.99 ±0.08	0.785
Systolic blood pressure, mm Hg	137.0 ±20.8	133.3 ±11.6	0.659
Diastolic blood pressure, mm Hg	88.3 ±12.9	88.4 ±4.7	0.993
Fasting glucose level, mmol/L	5.83 ±0.43	6.18 ±0.65	0.217
OGTT 2-h plasma glucose, mmol/L	7.93 ±2.67	7.18 ±2.46	0.554
Fasting insulin level, uU/mL	11.67 ±4.05	12.32 ±7.79	0.828
HOMA-IR	3.07 ±1.22	3.41 ±2.33	0.702
Haemoglobin A1c, %	5.64 ±0.30	5.71 ±0.38	0.688
Total Cholesterol, mmol/L	4.82 ±0.68	5.19 ±1.18	0.438
HDL, mmol/L	0.92 ±0.31	1.16 ±0.17	0.080
TG, mmol/L	2.22 ±1.14	1.76 ±0.83	0.357
Fat mass, kg	33.2 ±9.2	33.5 ±8.9	0.948
Lean mass, kg	58.1 ±21.9	60.7 ±14.0	0.781
Fiber, g/day	25.2 ±13.0	19.7 ±8.3	0.323
Fiber, g/1000 kcal	10.3 ±2.6	10.3 ±3.8	0.982
Stool frequency, x/day	2.00 [0.00, 4.00]	1.00 [1.00, 3.00]	0.346
Bristol Stool Form Scale, unit	4.33 ±1.58	3.56 ±1.35	0.300
Observed richness	121.7 ±17.7	94.8 ±19.7	0.010
Shannon diversity index	3.92 ±0.27	3.58 ±0.52	0.111
Pilou’s evenness index	0.82 ±0.04	0.79 ±0.08	0.346
Phylogenetic diversity	12.51 ±1.75	11.12 ±1.93	0.139

Averages are represented as mean (SD), except for stool frequency (median [IQR])

Table S15. Differences in changes (12 weeks compared to baseline) in metabolic health markers, fecal and plasma short-chain fatty acids, anthropometric, and bowel function outcomes in low compared to high responders based on peripheral insulin sensitivity.

	**Low Responders**	**High Responders**	**p-value**
HDL-cholesterol (mmol/L) change	-0.04 (0.01)	0.07 (0.04)	0.019
Triglycerides (mmo/L) change	-0.01 (0.08)	-0.54 (0.22)	0.046
Small adipocytes (%) change	0.84 (2.17)	7.51 (1.81)	0.033
Very large adipocytes (%) change	-0.60 (2.33)	-6.13 (2.35)	0.115
Fecal butyrate (mmol/kg) change	0.19 (0.34)	1.85 (0.40)	0.006
Fecal propionate (mmol/kg) change	0.41 (0.32)	2.23 (0.67)	0.031
Fecal acetate (mmol/kg) change	8.64 (2.39)	10.24 (2.00)	0.614
Plasma acetate (µmol/L) change	-14.12 (13)	17.94 (12.56)	0.097
Plasma propionate (µmol/L) change	0.17 (0.2)	0.51 (0.73)	0.665
Plasma butyrate (µmol/L) change	0.26 (0.15)	0 (0.14)	0.229
Plasma acetate % change	-10.88 (11.10)	36.69 (18.34)	0.045
Plasma propionate % change	3.19 (4.59)	79.53 (59.24)	0.234
Plasma butyrate % change	25.14 (11.96)	13.01 (16.24)	0.557
BMI (kg/m^2) change	-0.26 (0.16)	-0.38 (0.33)	0.759
Weight (kg) change	-0.75 (0.30)	-1.21 (0.80)	0.601
Stool softness (BSFS) week 12	4.75 (0.68)	4.00 (0.65)	0.435
Stool softness (BSFS) Change	1.19 (0.72)	-0.33 (0.99)	0.233
Stool frequency (x/day) week 12	2.00 [1.00, 3.35]	2.00 [1.00, 2.50]	0.580
Stool frequency (x/day) Change	0.50 [0.00, 1.25]	0.00 [0.00, 0.00]	0.085

Averages are represented as mean (SEM), except for stool frequency (median [IQR])

.
